# Characterisation of hydraulic properties of commercial gas diffusion layers: Toray, SGL, MGL, woven carbon cloth

**DOI:** 10.1038/s41598-024-68681-4

**Published:** 2024-08-13

**Authors:** Grace Esu-Ejemot Aquah, Daniel Niblett, Javad Shokri, Vahid Niasar

**Affiliations:** 1 Department of Chemical Engineering, University of Manchester, Manchester, M13 9PL UK; 2 School of Engineering, Newcastle University, Newcastle upon Tyne, NE1 7RU UK

**Keywords:** Gas diffusion layer, Hydraulic permeability, Computational fluid dynamics, Pore-scale simulation, Fuel cells, Fuel cells, Porous materials

## Abstract

This study utilises computational fluid dynamics simulations with the *OpenFOAM* computational framework to investigate and compare the in-plane and through-plane permeability properties of four different gas diffusion layers (GDLs). Also the through-plane water and air relative permeability values and water saturations at different rates were simulated. Permeability analysis enhances our understanding of fluid flow, ways to decrease pressure loss in the GDL, and methods to enhance oxygen concentration at the catalyst layer interface through convection. The analysis reveals that the investigated GDL materials have spatial heterogeneity of porosity and permeability, especially in the Sigracet SGL 25 BA GDL. However, the porosity and permeability of the Toray TGP-H 060 and AvCarb 370 MGL GDLs exhibit less variations. The two-phase flow studies on GDL saturation show that at the same water injection flowrate, the AvCarb 370 MGL GDL has the largest remaining water saturation, with Sigracet SGL 25 BA GDL being the less saturated GDL among the four investigated GDLs. The compression from the ribs significantly affected the in-plane permeabilities of both Toray TGP-H 060 and especially impacted Sigracet SGL 25 BA GDL. This impact was expected as the pore size distribution varied significantly in the areas under the ribs versus the channel.

## Introduction

### Gas diffusion layer (GDL)

Proton Exchange Membrane Fuel Cells (PEMFC) utilise hydrogen or alternative fuels to undergo electrochemical reactions with oxygen, facilitated by catalysts, resulting in electricity generation, with water and heat as byproducts^1^. They offer several advantages over other fuel cell types, including continuous energy production, simple structural design, rapid startup, extended operational longevity, and potential for providing clean energy solutions with high energy density^2,3^.

The PEMFCs relies on various porous materials, with the gas diffusion layer (GDL) playing a pivotal role in fluid distribution to and from the catalyst layers. The GDL serves as a porous electrode support, located between the catalyst layer and the bipolar plates, providing structural support and safeguarding the catalyst and membrane^4,5^. It facilitates the transport of immiscible fluids, water and air in PEMFCs, via its void space^6^. Thus, the permeability field in the GDL is a crucial factor to facilitates movements of both water and gas with preferably separate pathways for water and gas.

The commercially-available GDLs, mostly consisting of only a macro-porous substrate (MPS)^7^, include carbon fibre cloths or carbon fibre papers with a thickness of 180 to 500 $\mu$m^5,8,9^ bound together with a polymeric binder^10^. In terms of characterisation, the physical structure of the GDL has been analysed using ex-situ properties such as the distribution of pore and fibre sizes, porosity, compressibility, tortuosity, and GDL thickness^2,7^. Each of these properties, in turn, affects the hydraulic properties of the GDL, which are the fundamental properties of the GDL that determine fuel cell performance.

**Figure 1:**
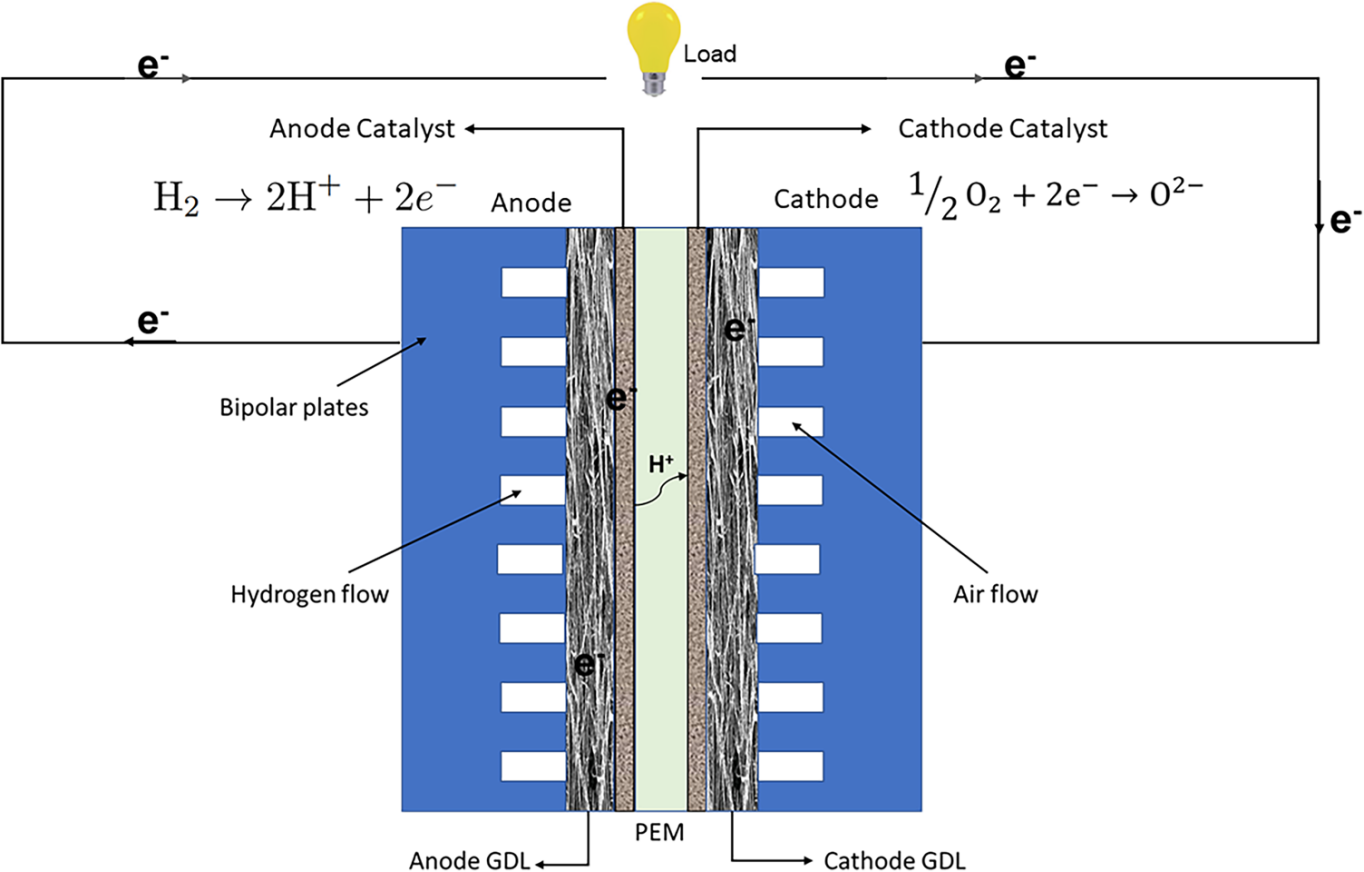
Schematic of a proton exchange membrane fuel cell (PEMFC) and its operating principle. Half-cell reactions have been shown for the anode and the cathode. PEM: Proton Exchange membrane, $e^-$: Electron, $H^{+}$: Proton.

In hydrogen PEMFC as illustrated in Fig. 1, humidified hydrogen and oxygen diffuse from the anode and cathode flow channels through the respective GDL, respectively, towards the catalyst layer where the cell reaction occurs. The full cell reaction is provided in the following equation, and half-cell reactions have been shown in Figure 1:

1 $$\begin{aligned} {\textrm{ H}}_2 + \frac{1}{2}{\textrm{O}}_2 \rightarrow {\textrm{H}}_2 {{\rm O }+ {\rm heat} + {\rm electricity}}, \Delta G_f^c ({\textrm{H}}_{\textrm{2}}{\textrm{O}})=-237.2 {\textrm{kJmol}}^{-1} \end{aligned}$$

Water is the by-product of electricity production in PEMFC, as shown in Eq. (1). At high current density, if not effectively removed, it could restrict the transport of the gas phase to the cathode catalysis layer (CL) - a phenomenon known as water flooding.

Furthermore, the membrane in the PEMFC must be sufficiently hydrated during operation to enable highly conductive ion pathways in the ionomer and membrane, and thus, over-drying should be avoided. Although the water produced at the interface between the cathode catalyst and the membrane might hydrate the membrane, it is necessary to maintain a balance between membrane hydration and the amount of water at the catalyst-membrane interface in order to prevent hindered transport of reactant gas. This water management issue is the major challenge of the PEMFC and PEMWE. Therefore, optimising the efficient transport of species into and out of the electrochemical device at elevated current densities is likely to improve its performance^6^. As this process is mainly attributed to the permeability of the GDL, among other factors, it has become one of the most recognised parts of the fuel cell in need of improvement and constant optimisation^7^.

### Flow properties of GDLs

One way of significantly improving and optimising the PEM fuel cells or electrolysers’ performance is to reduce the mass transfer loss in GDLs, which is directly controlled by the flow properties of GDLs. Permeability, a hydraulic metric characterising mass transfer in fibrous porous media, is a key parameter that denotes the fluid flow within a porous material^11^. In the GDL, the absolute permeability has been shown to indicate the GDL’s capacity to support convection-driven mass transfer^2^ and could lead to better cell performance^12^ while the relative permeability is crucial in two-phase flow as it affects the transport and distribution of liquid water saturation^13^.

Multiple research studies have investigated the absolute and relative permeability of GDLs in both in-plane and through-plane directions either experimentally^2,14–19^, numerically including pore-network models PNM^20,21^, lattice Boltzman method (LBM)^22–27^, and other computational fluid dynamics (CFD) methods^28,29^, or analytically^30–33^. Furthermore, within the three-dimensional fibrous structure, permeability is intricately linked to both the void volume fraction (porosity) and the three-dimensional orientation of fibres (tortuosity)^34^ and also the fibre size^2,18^; thus, a reliable model should incorporate these factors. While some experimental approaches established correlations between GDL permeability and other hydraulic properties such as porosity, pores structure, PTFE loading, and GDL thickness^2,15,19^, numerical and analytical models were mainly used to calculate these hydraulic properties^35,36^. For instance, to address the impact of fibres’ orientation, an analytical method was employed by Tamayol and Bahram^32^. They arranged fibres of similar dimensions parallel and perpendicular to the flow direction to mimic the GDL microstructure. Thereafter, the permeability of parallel fibres and those of perpendicular fibres were calculated. The overall estimated GDL permeability was derived by combining the permeability of both fibre groups. However, despite its theoretical foundation, this method inadequately captures the intricate microstructure of the GDL, characterised by a stochastic distribution of fibres, especially due to the very thin thickness of the GDLs compared to the planar dimensions of GDLs^7^. Consequently, extensive investigations into the in-plane and through-plane permeability and relative permeability of realistic and commercially available GDLs become necessary when considering numerical or analytical methods. Numerical simulations, such as Lattice Boltzmann Method (LBM) and Navier-Stokes equations, have been instrumental in assessing these permeability characteristics^22,24–27^. These simulations often reconstruct GDL microstructures from micro-tomographic images or artificial fibre media, enabling accurate estimation of hydraulic parameters without the need for complex experiments. Modern X-ray imaging technology further facilitates high-resolution imaging and simulations, complementing experimental approaches^24,25^.

For example, Rama et al.^24^ integrated X-ray micro-tomography and LBM to investigate the anisotropic permeability of a carbon cloth GDL using X-ray shadow images^24^. Similarly, Jiang et al.^25^ utilised LBM in a stochastically-generated GDL to explore alterations in through-plane and in-plane permeability by manipulating parameters like porosity, fibre diameter, and pressure gradient^25^. Both studies successfully established correlations for permeability prediction, yielding values comparable to experimental data, highlighting the utility of numerical simulations in understanding GDL permeability characteristics. Limited literature exists on the relative permeability of air and water across gas diffusion layers (GDLs) due to challenges in maintaining an immobile water phase and regulating saturation^29^. Rosen et al. utilized the Lattice Boltzmann Method (LBM) to model flow in Toray TGP-H-60 material and compared results with experimental data^37^. Hao et al. conducted pore-scale LBM simulations on stochastically generated GDLs to investigate the impact of anisotropic characteristics on relative permeability^23^. Their findings suggest the flow direction has minimal influence on the relative permeabilities of wetting and non-wetting phases. Pore Network Models (PNM) have also been useful in calculating relative permeability in porous materials, including GDLs, despite oversimplification of pore morphologies in thin media^38^. However, few studies have specifically generated relative permeability curves for studied GDLs, despite their importance in understanding two-phase flow behaviours and optimising material design and operation^29,30,38,39^. These curves offer insights into the influence of pore size distribution on fluid transport and mass transfer loss, therefore, electrochemical reactant conversion performance. Notably, no comparative analysis of absolute and relative permeability curves across different commercial GDLs has been conducted, emphasising the need for such a study^29,30^.

### This study

In this study, we aim to address the shortcomings in former studies, as follows:

- Unlike pore-network models, we solve the flow inside the commercial GDLs using full CFD simulation, implemented by the VOF method in *OpenFOAM*. Since in this method, we do not need to simplify the geometrical topology, the impact of oversimplification of pore morphology will be eliminated. Former studies show that significant error is expected in pore-network models applied to high porosity medium^40,41^. Furthermore, our model allows for the pore-scale simulation of the GDL reconstructed from tomographic images considering the binders and PTFE^33,42^. Consequently, we conduct a comparative analysis of the permeability in various commercial GDLs with respect to in-plane and through-plane directions in a single phase.
- We quantify the impact of GDL compression on permeability and the change of pore-size distribution before and after compression under ribs. This is important to give insights into the water accumulation under the ribs and the potential for gas invasion for hydrophilic pores.
- In relative permeability curves using for two-phase flow Darcy’s law, two major aspects are critically important, end-point saturation and curvature of the relative permeability curves. The end-point saturation is the maximum level of saturation reached by a porous media with a particular substance such as air or water. In high porosity systems such as GDLs, it is expected that a mild curvature (almost linear) relation between relative permeability and saturation will be observed. To understand better the end-point saturation at different flow rates, we investigate the end-point relative permeability data, which are essential for developing volume-average-based models such as Darcy-based flow simulators using the PEMFC or PEM electrolyser simulations^43,44^. We have focused specifically on X-ray computed tomography data from four common commercial GDLs as they are widely used, and we can provide a consistent analysis of their performance.

## Methods

X-ray computed tomography images of four different GDL materials were analysed in this study using simulations of single-phase and two-phase flow using the *OpenFOAM* computational framework. These simulations allowed for the determination of both the in-plane and through-plane absolute permeability, as well as estimation of end-point saturations of the relative permeabilities at different flow rates.

### GDL materials

Microtomography X-ray ($\mu$XRCT) images of commercial carbon papers, AvCarb MGL 370^45^ and SGL 25 BA^46,47^ with 5% PTFE coating, Toray TGP-H 060 with 10 % PTFE coating^48,49^ as well as a woven carbon fibre cloth^27^ have been considered for this simulation study.

To obtain the $\mu$XRCT of MGL, Heliscan micro-CT scanner was used with imaging parameters as follows: 26 keV photon source energy with an exposure time of 900 ms, and 2880 collected projections per sample^50^. The segmented version of this GDL was used in our studies, and more details about it can be found in^51^. For the woven, the $\mu$XRCT image was taken using the Zeiss Xradia Versa 620 X-ray Microscope at high resolution and also at low resolution. The final image used was super resolved using DualEDSR. Details about this can be found in^27^. X-ray imaging for Sigracet SGL 25 BA and Toray TGP-H 060 were performed at the TOMCAT beamline of the Swiss Light Source (SLS) with a GigaFRoST high-speed camera^48^. Toray TGP-H 060 had an exposure time per projection of 0.83 ms and 301 projections with a constant operation current of 0.62 mA. Further details can be found in^48^. In the case of the SGL, the GDL was oriented at a 90-degree angle to the beam. Exposure time was 3 ms per projection with a total of 251 projections^46^.

**Table 1:** Commercial GDL samples and specification of original images.

Manufacturer	Type	GDL description	Image description	Original image size	Voxel size ($\upmu$m)	Ref
Toray	TGP-H-060	Carbon paper	Oprando with channel	400 $\times$ 1001 $\times$ 194	2.75	^48,49^
AvCarb	MGL 370	Carbon paper	Dry unsegmented GDL	1800 $\times$ 1794 $\times$ 151	2.05	^45^
Sigracet	SGL 25 BA	Carbon paper	Operando with channel	767 $\times$ 1500 $\times$ 156	3.0	^46,47^
CeTech	W1S1011	Woven carbon cloth	Fused in MEA	4000 $\times$ 1100 $\times$ 3112	0.7	^27^

**Figure 2:**
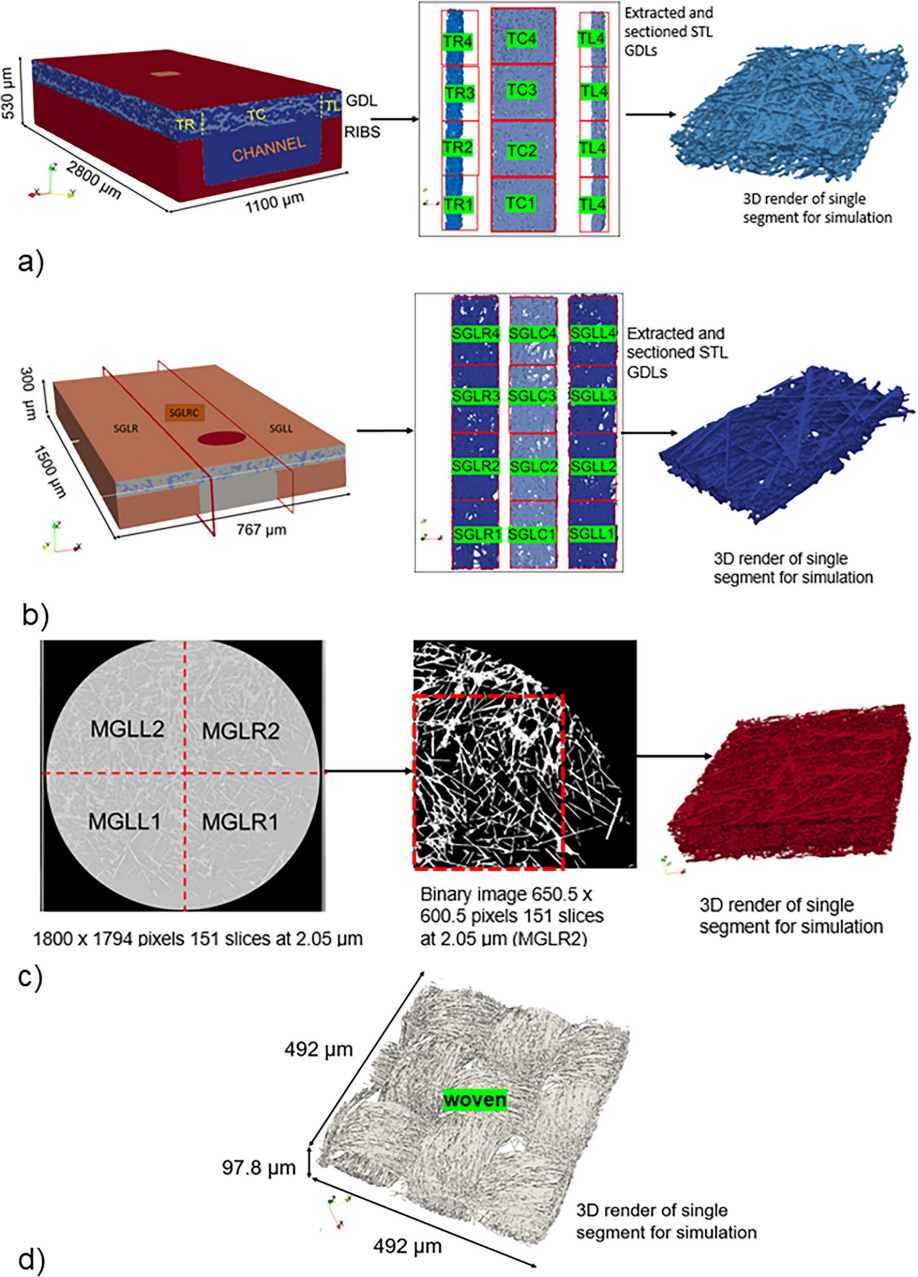
Visualisation of the microtomography X-ray images of (**a**) Toray TGP H-060 showing sections of regions under the channel (TC1–TC4) and the ribs to the left side (TL1–TL4) and right side (TR1–TR4) (**b**) Sigracet SGL 25 BA GDL showing regions under the channel (SGLC1–SGLC4) and the ribs (SGLL1–SGLL4, SGLR1–SGLR4) (**c**) AvCarb MGL 370 sectioned into MGL left (L1–L2) and MGL right (R1–R2) and (**d**) the (woven) carbon cloth.

The imaging processing of the Toray TGP-H 060 and SGL 25 BA was performed while the fuel cell was in operation. Figure 2 shows visually these four different GDL materials, while their corresponding description can be found in Table 1.

### Simulation domain

#### Toray TGP-H 060

A single GDL slice comprising total areas under the rib and under the channel had dimensions 400 $\times$ 1001 with an isometric voxel length of 2.75 $\upmu$m. To reduce the computational costs of the simulated domain, the GDL was divided into 12 sections for simulation purposes, as shown in Fig. 2a. Whether the GDL lies beneath the ribs or the channel affects its thickness. Thus, the regions located beneath the ribs and channels were partitioned to investigate the impact of compression on the permeability and porosity of the GDL. Four of these were areas under the channel (TC1–TC4), and eight sections were areas under the ribs to the left side (TL1–TL4) and right side (TR1–TR4).

#### Sigracet SGL 25 BA:

The slice size of the acquired $\mu$XRCT images of sigracet SGL 25 BA was 767 $\times$ 1500 with an isometric voxel size of 3 $\upmu$m^46,47^. Similar to the Toray GDL, the GDL in question was affixed to the bipolar plate, thereby encompassing regions beneath the ribs as well as regions beneath the channel. Subsequently, the surface was partitioned into compressed GDL regions (located beneath the ribs) and uncompressed GDL regions (located beneath the channel, as depicted in Fig. 2b). Four uncompressed parts, namely SGLC1, SGLC2, SGLC3, and SGLC4, with thicknesses of 168 $\upmu$m, four compressed parts on the left side, denoted as SGLL1, SGLL2, SGLL3, and SGLL4, with thicknesses of 135 $\upmu$m and four compressed parts on the right side, referred to as SGLR1, SGLR2, SGLR3, and SGLR4, with thicknesses of 135 $\upmu$ m.

#### AvCarb MGL 370

The acquired $\upmu$XRCT image of AvCarb MGL 370 GDL was circular with a slice size of 1800 $\times$ 1794 and an isometric voxel length of 2.05 $\upmu$m^45^. For this research, this circular GDL was divided into four and a square was removed from each quadrant for simulation, as seen in Fig. 2c. Four sections, MGLL1, MGLR1, MGLL2, and MGLR2, were obtained.

#### Woven carbon cloth

The woven GDL used in this study, W1S1011 CeTech, was obtained from a commercially prepared membrane electrode assembly of 25 $\hbox {cm}^2$. The membrane electrode assembly consisted of two GDLs made of woven carbon fibre cloth equipped with hydrophobic MPLs. More details can be found in^27^. We segmented out one GDL from the MEA. Thereafter, a single section of the GDL, 97.86 $\upmu$m thick and without the MPL, as illustrated in Fig. 2d, was utilised for the simulation.

Details of each section of the respective GDL materials are provided in Table S1 in the Supplementary Information (SI). Furthermore, for all of these GDL materials, the rendered images were converted to a stereolithography (STL) file and exported to snappyHexMesh for the meshing to be used for *OpenFOAM* simulations.

### Porosity, fibre and pore size distribution analysis

Porosity is a measure of voids present in a medium. It measures the volume of these voids in relation to the total volume of the medium. Pore size distribution and fibre size distribution, on the other hand, can help us to understand better the hydraulic properties of materials such as relative permeability^52^. The porosity of each section of all four GDL materials $\mathrm {\frac{V_{void}}{V_{total}}}$, was calculated, where V is the volume. Furthermore, the fibre size distribution of two GDL studied—TGP-H 060, and SGL 25 BA, was performed using a source code developed by^53,54^ in MATLAB, and the pore size distribution was analysed using the AVIZO 2019 software by calculating the equivalent spherical diameter ($D_{eq}$). Pore size has been defined by the largest inscribed sphere in a pore. Subsequently, the equivalent diameter is the diameter of a spherical particle of the same volume ($V_p$); $D_{eq} = \root 3 \of {\frac{6 V_p}{\pi }}$.

### Single-phase flow modelling

The Stokes equation was solved for the single-phase flow of air through the 3D porous GDL in x, y, and z directions using simpleFoam^55^ with absolute convergence criteria of $1\times 10^{-6}$. SimpleFoam is a steady-state solver which uses the SIMPLEC (Semi-Implicit Method for Pressure Linked Equations Consistent) algorithm to solve incompressible, turbulent flow. However, it can also be used to solve laminar flow. The specific equations solved in simpleFoam are conservation equations of mass and momentum,

2 $$\begin{aligned} \nabla \cdot \textbf{u} = 0, \end{aligned}$$

3 $$\begin{aligned} \nabla \cdot (\textbf{u u}) - \nabla \cdot (\nu \nabla \textbf{u})=-\nabla p, \end{aligned}$$

where **u**, $\nu$, and *p* denote velocity ($\mathrm {m\ s^{-1}}$), kinematic viscosity ($\mathrm {m^2\ s^{-1}}$) and Kinematic pressure ($\mathrm {m^2\ s^{-2}}$), respectively. Air was assumed incompressible since the pressure drop across the GDL was relatively small, and its flow was restricted to the void space. It is important to note that the pressure utilised in simpleFoam must be multiplied by the density in order to obtain the actual pressure in Pascal units.

### Meshing and mesh sensitivity analysis

Each GDL in the section above was scaled from voxel coordinates to real coordinates based on their respective resolutions, and the mesh was generated using *snappyHexMesh* in *OpenFOAM*. The grid dependency analysis was performed for the GDLs as presented in the Figure S1. The standard minimum angle and aspect ratio criteria were used to generate five different meshes with elements ranging from 500,000 to 7 million. *checkMesh* resulted in acceptable output with fewer than 4.5 $\times 10^{-5}\%$ skewed elements whose presence did not affect the simulation accuracy. Following the grid dependency analysis, it was determined that the simulation could reliably utilise grid cells numbering between 1.5 million and 5 million. The selection of mesh sizes was predicated on maintaining a permeability variation of less than 5%, ensuring the independence of results from mesh size while concurrently minimising computational time.

### Two-phase flow modelling

The immiscible and incompressible two-phase flow simulation in the GDL was conducted using the Volume of Fluid (VoF) method. This method, which Hirt and Nicholas first introduced^56^, uses the volume fractions of each fluid phase to obtain the average density and viscosity to be used in the mass and momentum conservation laws. The conservation laws are amended by an interface transport equation as a result of the modification by Brackbill using the Continuum Surface Force (CSF) model^57,58^.

Using *interFoam* solver in *OpenFOAM*, we solved the coupled continuity equations (4) and Navier-Stokes (5) to get the pressure and velocity.

4 $$\begin{aligned} \nabla \cdot \textbf{u}= 0 \end{aligned}$$

5 $$\begin{aligned} \frac{\partial }{\partial t} (\rho \textbf{u})+\nabla \cdot (\rho \textbf{u u})=-\nabla p+\nabla \cdot \left[ \mu \left( \nabla \textbf{u}+\nabla \textbf{u}^T\right) \right] + \rho \textbf{g} + F_\sigma \end{aligned}$$

The volume-averaged dynamic viscosity ($\mu$ with unit Pa s) and density ($\rho$ $\mathrm{with unit kg \, m}^{-3}$) were calculated from the liquid phase fraction ($\alpha$) as $\mu = \mu _l \alpha + \mu _g(1 - \alpha )$ and $\rho = \rho _l \alpha + \rho _g(1 - \alpha )$. Subscripts *l* and *g* are the liquid and gaseous phases, respectively. Here, the liquid is water ($\alpha$ = 1), and the gas is air ($\alpha$ = 0). $F_{\sigma }$ is the volumetric interfacial force and is defined by;

6 $$\begin{aligned} {F_\sigma =\sigma \kappa (\alpha ) \nabla \alpha } \end{aligned}$$

with interface curvature ${\kappa =-\nabla \cdot \left( \frac{\nabla \alpha }{|\nabla \alpha |} \cdot \textbf{S}_{\textbf{f}}\right) }$, where ${\mathbf {S_f}}$ is the cell face. The transport of the volume fraction $\alpha$, is achieved using

7 $$\begin{aligned} {\frac{\partial \alpha }{\partial t}+\nabla \cdot (\alpha \textbf{u}) + \nabla \cdot (\alpha (1-\alpha )\textbf{u}_r)=0}, \end{aligned}$$

where $\textbf{u}_r$ is the relative velocity between the two fluids and is given by $\textbf{u}_l-\textbf{u}_g$. The time step ($\Delta$t) for the simulations was selected by choosing a Courant number, denoted as $Co = \frac{\Delta t U}{\Delta x} < 0.5$ where *U* denotes the velocity magnitude, and $\Delta x$ is the cell size.

### Boundary, initial and operational conditions

**Figure 3:**
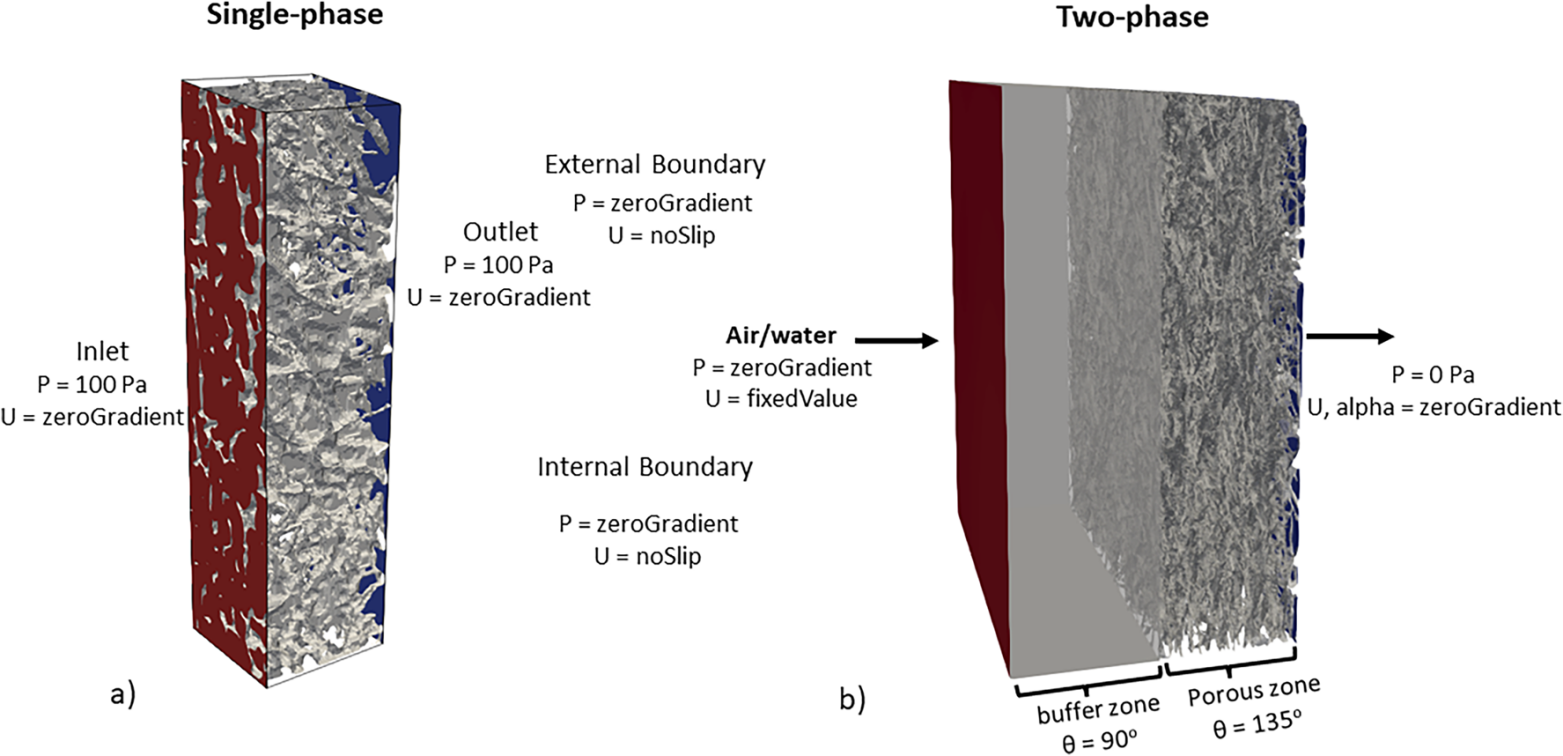
Boundary conditions for the (**a**) single-phase flow simulations, and (**b**) two-phase flow simulations. In air (water) simulation, the buffer zone is saturated with air (water) and the porous zone is saturated with water (air).

Figure 3 illustrates the boundary conditions employed in this study. For the single-phase flow simulations, air was injected into the domain at a constant inlet pressure of 100 Pa, and the outlet pressure was set to zero. At the same time, zero gradients were imposed on the internal (void-solid interface) and external boundaries for pressure. The velocity component was subjected to zero gradient boundary conditions at the inlet and outlet. In contrast, the external boundaries and the void-solid interface were subjected to the no-slip boundary condition.

For the two-phase flow simulations, a fixed velocity inlet boundary condition was applied. Pressure was kept constant in the outlet. Similar to the single-phase case, the no-slip boundary condition was applied to the domain walls and void-solid interfaces. Given capillary forces control the invasion into the porous medium and to avoid the boundary condition effects on invasion, a buffer zone was introduced with a thickness equal to the thickness of the respective porous GDL zone. It is assumed that the GDL surfaces have contact angles (θ) of 135°, while the buffer walls were maintained at a contact angle of 90°. In such conditions water is considered as the non-wetting phase, whereas air is the wetting phase.

The fluids were assumed to be at the temperature of $70 ^\circ$C. The capillary numbers of air and water in a functioning PEM fuel cell were approximated by utilising the current density data acquired from a previous study^59^. The fuel cell was working at a temperature of $70 ^\circ$C. The authors did not report the type of GDL used. It is assumed that a comparable pattern will be observed in the polarisation curve at a specific operating condition. Based on the provided current density data, the maximum capillary number observed in an operating fuel cell was around $1.29\times 10^{-6}$ for air and $2.61 \times 10^{-14}$ for water. Considering how low the values are and to save computational time, we used uniform capillary numbers of $6.27\times 10^{-3}$, $6.27\times 10^{-4}$, and $6.27\times 10^{-5}$ corresponding to 1 $\textrm{m\, s}^{-1}$, 0.1 $\textrm{m s}^{-1}$, and 0.01 $\textrm{m s}^{-1}$ velocities to inject the invading fluid for the two-phase flow simulations. Table 2 shows other parameters used for the calculation.

**Table 2:** Physical properties used for the numerical simulations at $70 ^\circ$C.

Parameters	Value	Unit
Kinematic viscosity of air	$1.995 \times 10^{-5}$	$\textrm{m}^{2}$ $\textrm{s}^{-1}$
Air–water interfacial tension	0.0644	$\textrm{N m}^{-1}$
Density of air	1.029	$\textrm{kg m}^{-3}$
Kinematic viscosity of water	$4.13 \times 10^{-7}$	$\textrm{m}^{2}$ $\textrm{s}^{-1}$
Density of water	977.76	$\textrm{kg m}^{-3}$

### Upscaled absolute permeability and end-point relative permeability of GDLs

The results of the pressure and velocity fields generated from the solution of the equations were upscaled to calculate the absolute and relative permeabilities of the GDLs. Darcy’s law is the volume-averaging equation commonly used to capture the flow of single or two fluid phases in a porous medium. Similarly, we used Eq. (8) to calculate the absolute permeability in through-plane and in-plane directions, $K_i$.

8 $$\begin{aligned} K_i = \mu \left( \frac{P_{in} - P_{out}}{L}\right) ^{-1} \left( \frac{1}{A} \int _{A} u_i\,dA\ \right) , \end{aligned}$$

where *i* represents *x*, *y*, or *z* direction, $\mu$ is the dynamic viscosity (Pa s), *P* is the average pressure at the inlet or outlet of the simulation domain (Pa), L is the length from inlet to outlet where average pressure values have been calculated (m), A is the cross-sectional area of domain normal to flow ($\textrm{m}^2$), and *u* is the velocity along the direction *i* ($\text {m}^{-1}$). In-plane (*x*- and *y*-directions) and through-plane (*z*-direction) permeability were calculated for each section of the GDLs.

For the upscaled two-phase flow in porous media, two-phase Darcy’s law was used in which the relative permeability functions are introduced as a nonlinear function of average fluid saturations. Equation (9) shows Darcy’s law for two-phase flow in a porous medium.

9 $$\begin{aligned} \mathbf {u^\alpha } = - \frac{k_r^\alpha (S^\alpha ) \textbf{K}}{\mu ^\alpha } \left( \nabla P^\alpha - \rho ^\alpha \textbf{g} \right) , \end{aligned}$$

in which $k_r^\alpha (S^\alpha )$ is the nonlinear function relative permeability as a function of phase saturation.

However, for the GDL applications in which the thickness of the domain is much smaller than the other dimensions of the GDLs, we are interested in two-phase flow through the layers at Darcy scale, and gravity effect on two-phase flow can be ignored. Thus, Eq. (9) was simplified to Eq. (10):

10 $$\begin{aligned} u_z^\alpha = - \frac{k_r^\alpha (S^\alpha ) K_z}{\mu ^\alpha } \frac{\partial P}{\partial z}, \end{aligned}$$

where $u_z^\alpha$ is the Darcy velocity of fluid phase $\alpha$ through the GDL, $\partial P/\partial z$ is the pressure gradient over the GDL thickness.

### Method validation

#### Single-phase Poiseuille flow simulation

To assess the computational accuracy of the method, a cylindrical Poiseuille flow was chosen to validate the simulation. The domain was a two-dimensional pipe with a length (L) of 600 microns and a height (h) of 60 microns. Hence, using Eq. (11), which is modified form of Hagen-Poiseuille equation for a rectangular channel, one can determine the velocity profile and maximum fluid velocity using the same pressure drop and kinematic viscosity listed above. The flow was considered to be a laminar flow, incompressible, and occurs at steady-state condition. The results of the calculation are displayed in Fig. S2 . The simulation results align with the analytical solution, demonstrating that the solver’s boundary format accuracy and stability meet the necessary criteria.

11 $$\begin{aligned} u(y) = \frac{\Delta P}{2 \mu L} \left( \frac{h^2}{4} - y^2 \right) \end{aligned}$$

where y ranges from $-h/2$ to h/2

In addition, the Darcy equation is utilised to determine the permeability values. We obtained a value of K = $3.01 \times 10^{-10}$ $\textrm{m}^{2}$ using the OpenFoam, simpleFoam solver, while the analytical solution yielded a value of K = $3.00 \times 10^{-10}$ $\textrm{m}^{2}$.This shows that our method is capable of calculating the permeability with a 0.5 % error.

## Results and discussion

### Absolute permeability of GDLs

**Figure 4:**
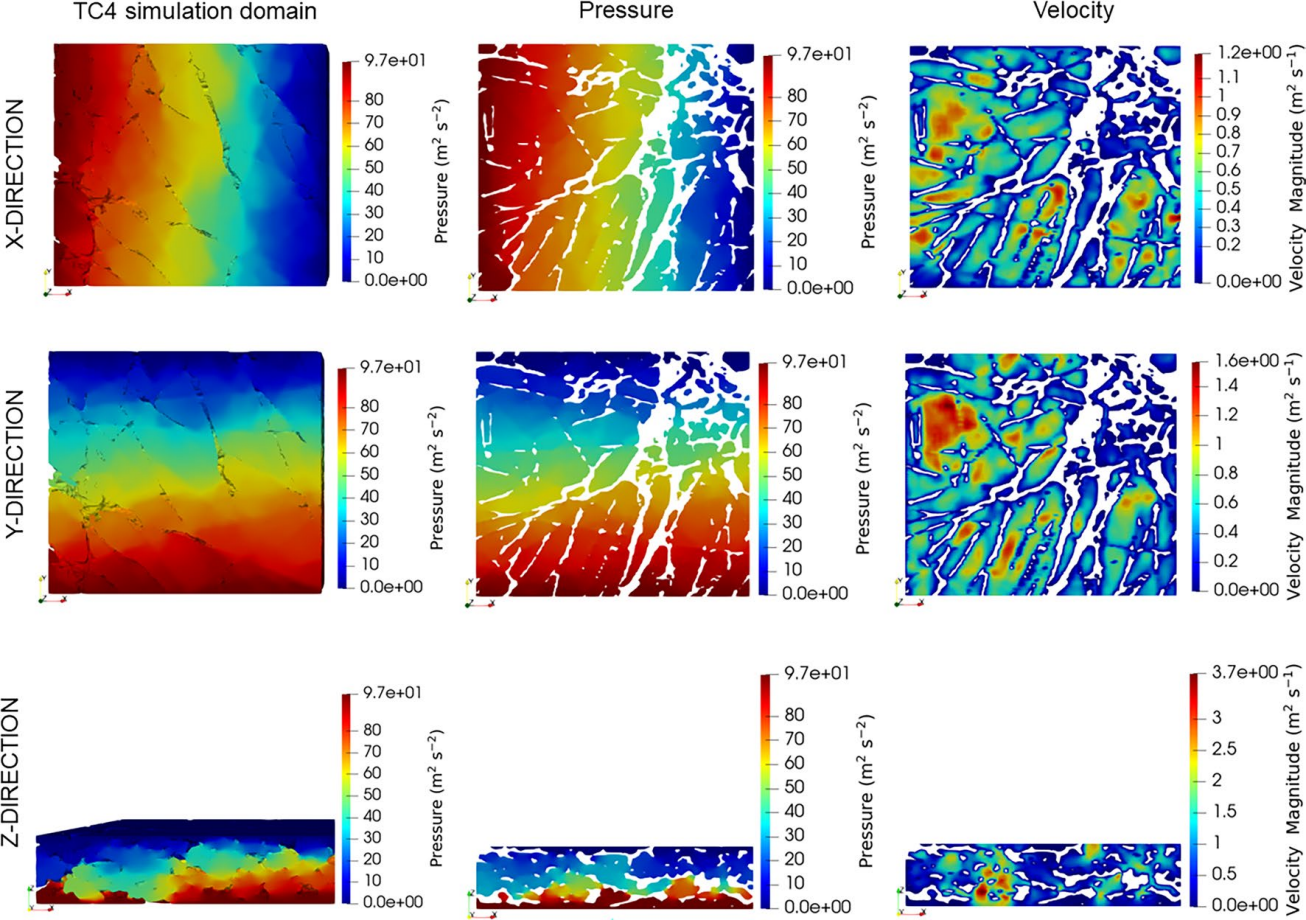
Pressure-velocity fields for Toray TGP-H 060 (TC4) are depicted: the first column displays the entire simulation domain, the second column exhibits a slice of the domain illustrating pressure field, and the third column presents a slice of the domain depicting the velocity field.

Velocity and pressure fields were calculated for each simulation domain and upscaled to calculate the respective permeability. Figure 4 shows the calculated fields for a section in the Toray GDL, TC4. The first column presents the complete simulation domain, which is a three-dimensional representation of the GDL pore space. It illustrates the pressure gradient in the x, y, and z directions. In addition, we present a cross-section of the domain to visualise the pressure and velocity distributions in the x, y, and z directions. These distributions are displayed in columns two and three, respectively. This visualisation validates the movement within the pore spaces of the material and demonstrates the interconnectedness of these pore spaces.

**Figure 5:**
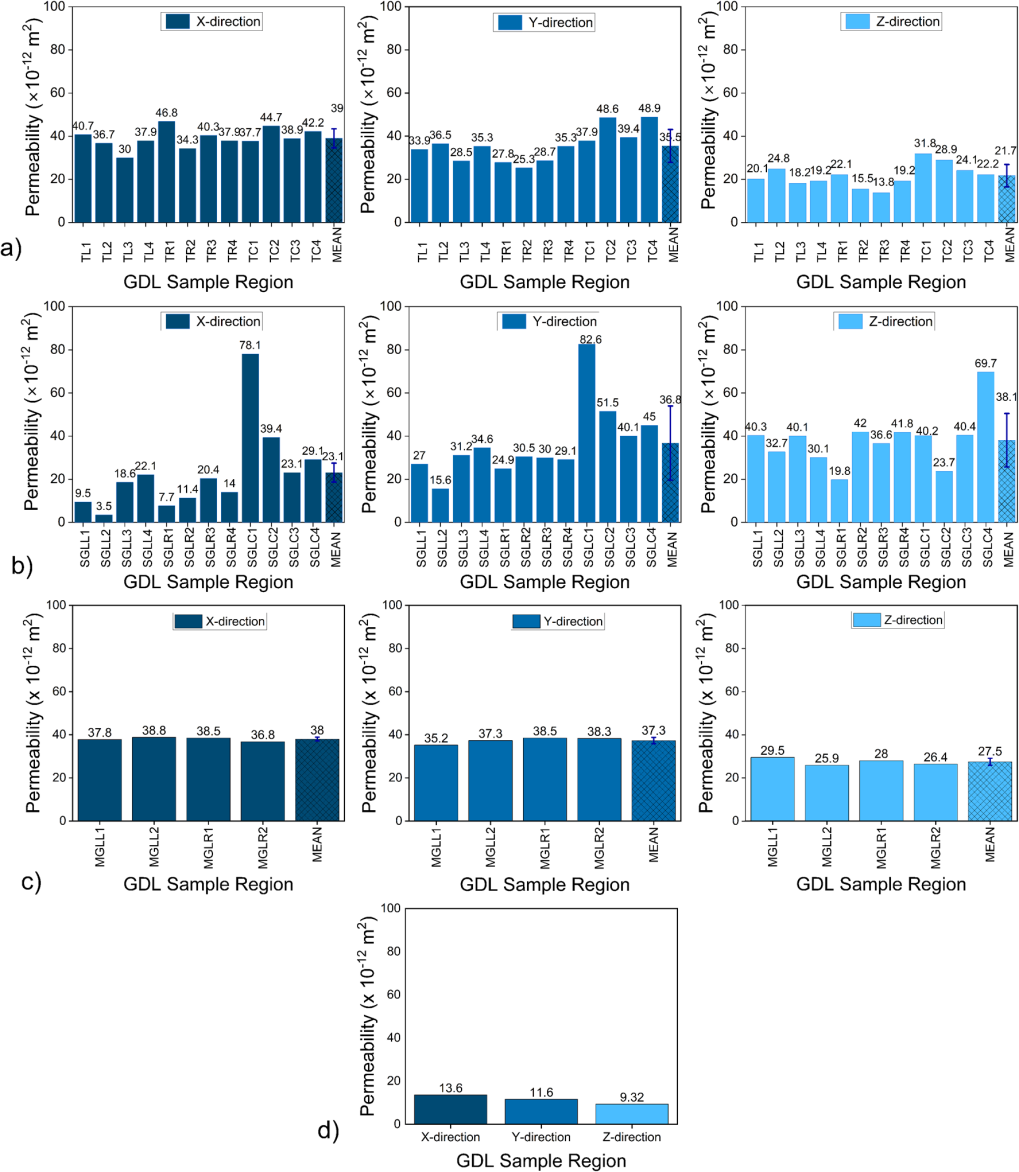
In-plane (x and y directions) and through-plane (z-direction) absolute permeability ($\textrm{m}^2$) of (**a**) Toray TGP-H 060 GDL, (**b**) SGL 25 BA GDL, (**c**) Avcarb MGL 370, and (**d**) the woven cloth.

The findings pertaining to the simulated absolute permeability of the GDLs are illustrated in Fig. 5. In each subplot, the last bar shows the mean permeability of the GDL samples in the x, y, and z directions. The accompanying error bar illustrates the standard deviation of the measured permeability values, providing a visual representation of the data variability around the mean. Figure 5a shows the results of the TGP-H GDL. The mean values of in-plane permeability in the x-direction and y-direction are 39.0 $\times 10^{-12}$ $\textrm{m}^2$ and 35.5 $\times 10^{-12}$ $\textrm{m}^2$, respectively, while the through-plane permeability is 21.7 $\times 10^{-12}$ $\textrm{m}^2$. The respective standard deviations for these measurements are 4.4 $\times 10^{-12}$ $\textrm{m}^2$ (x-direction), 7.6 $\times 10^{-12}$ $\textrm{m}^2$ (y-direction), and 5.0 $\times 10^{-12}$ $\textrm{m}^2$ (through-plane). These values are derived from permeability calculations for areas under both the ribs and the channel of the TGP-H GDL. The results indicate the expected variability in GDL permeability during actual operations, which may contribute to the uneven formation of water clusters during fuel cell operation.

Furthermore, the average permeability in the through-plane (z) direction is significantly lower than in the average in-plane (x and y) directions within this GDL. We noticed a 42 % decrease in the permeability of the through-plane direction compared to the average in-plane direction. This aligns with the conclusions drawn by other authors, e.g. While investigating Toray TGP-H-060 GDL with 20 % PTFE loading and a porosity of 0.6–0.75, the rough-plane permeability was calculated to be 6.6 $\times 10^{-12}$ $\textrm{m}^2$ while the in-plane permeability was 14.4 $\times 10^{-12}$ $\textrm{m}^2$^37^. This shows a 54% decrease in the permeability of the through-plane direction compared to that of the in-plane direction. It should be noted that factors like the PTFE concentration, thickness, and porosity could cause our permeability values to differ. However, the trend of a lower through-plane permeability aligns.

As illustrated in Fig. 5b, the mean permeability in the through-plane (z) direction of the SGL is 38.1 $\times 10^{-12}$ and exceeds that in the in-plane x and y directions which are 23.1 $\times 10^{-12}$ and 36.8 $\times 10^{-12}$ respectively. As in the case of TGP-H, these values are the mean results derived from the analysis of the twelve sections of the GDL. The standard deviations are 19.9 $\times 10^{-12}$, 17.2 $\times 10^{-12}$, and 12.4 $\times 10^{-12}$ for the x, y, and z directions respectively. The observed higher permeability in the through-plane direction can be attributed to the fibre arrangement, indicating that the fibres are not predominantly aligned in the usual in-plane direction, a common trait in most GDLs. This observation is supported by previous studies^60^, which suggested that fibre orientation within a porous material notably influences permeability, especially when the material’s porosity is below 0.7, as is the case with the SGL GDL with a calculated mean porosity of 0.65. Consequently, the higher through-plane permeability in the SGL may suggest that its fibres are not oriented parallel to that direction, as is typically observed in other GDLs such as Toray.

Furthermore, larger fibres arranged sparsely (see Fig. 7) can notably enhance flow channels within the material, thereby enhancing permeability along the fibre orientation. This enhancement occurs because binders present in sparsely arranged fibres can penetrate the pores more effectively, thereby impeding flow in the in-plane direction. This phenomenon becomes more pronounced with increased compression, elucidating the particularly low in-plane permeability observed under the ribs. To compare our results with literature, we used only the four areas under the channel with average porosity of 0.71. Here, the average through-plane permeability we calculated for SGL 25 BA yielded a value of 43.5 $\times 10^{-12}$ $\textrm{m}^2$, and is comparable to the permeability values reported in the literature for the commercial GDL 25 BA (45.4 $\times 10^{-12}$ $\textrm{m}^2$)^61,62^. In contrast, the permeability values for SGL 25 BA, calculated using the PNM method, were determined to be 14 $\times 10^{-12}$ $\textrm{m}^2$^21^. The discrepancy could be attributed to the simplification of the geometry, a common feature of the PNM.

The permeability values for MGL are displayed in Fig. 5c. Similarly to the TGP-H, the findings indicate that the permeability in the through-plane direction is comparatively lower than in the in-plane direction. The permeability exhibits similar features in the in-plane x and y directions. The mean permeability values for the GDL are 38.0 $\times 10^{-12}$ $\textrm{m}^2$, 37.3 $\times 10^{-12}$ $\textrm{m}^2$, and 27.5 $\times 10^{-12}$ $\textrm{m}^2$ in the x, y, and z directions, respectively. The corresponding standard deviations are 0.89 $\times 10^{-12}$ $\textrm{m}^2$, 1.5 $\times 10^{-12}$ $\textrm{m}^2$, and 1.62 $\times 10^{-12}$ $\textrm{m}^2$. These data indicate minimal variation within the GDL simulation sections used in this study. This lack of variability can be attributed to the absence of compaction effects from gaskets or ribs, which contrasts with the conditions experienced by other GDLs. Consequently, this GDL exhibits consistent permeability characteristics under the simulation conditions.

The permeability of the carbon cloth GDL is provided in Fig. 5d. The simulation utilised only one specific portion of the GDL material, comprising a total of 9 tows. The findings indicate that the permeability of the through-plane (z) direction is comparatively lower than those of the in-plane (x and y) directions. This contradicts previous findings^24^, where the through-plane permeability of a carbon cloth GDL was four times greater than its in-plane permeability. Here, the authors reported values of $23.5 \times 10^{-12}$ $\text {m}^2$, 5.3 $\times 10^{-12}$ $\text {m}^2$, and $6.5\times 10^{-12}$ $\textrm{m}^2$, for the through-plane and in-plane x and y directions respectively. However, our findings, as seen in Table 3, align closely with the permeability measurements of 14.5 $\times 10^{-12}$ $\textrm{m}^2$ for in-plane permeability reported by the authors that sourced this GDL^27^. In the former, the authors suggested that the significant rise in their values could be attributed to fibre scattering resulting from the trimming of the GDL tows^24^, while our study had no such limitation. In addition to their suggestion regarding the notably high permeability values in the through-plane direction, we propose that the carbon cloth’s Representative Elementary Volume (REV) size could have influenced their simulation results. Their approach involved dividing a GDL comprising 12 weaves into 21 segments for simulation using the Lattice Boltzmann Method (LBM). Given that the pore size between the fibre tows is relatively large, while inside the tows, it is small, it follows that if a domain predominantly covers the macro-pore size, permeability will be high, whereas if it covers only the tow, permeability will be small. Our GDL’s REV consists of 9 weaves, and we utilised it as a single domain, thereby addressing this potential limitation of the previous study^63^.

### Porosity

**Figure 6:**
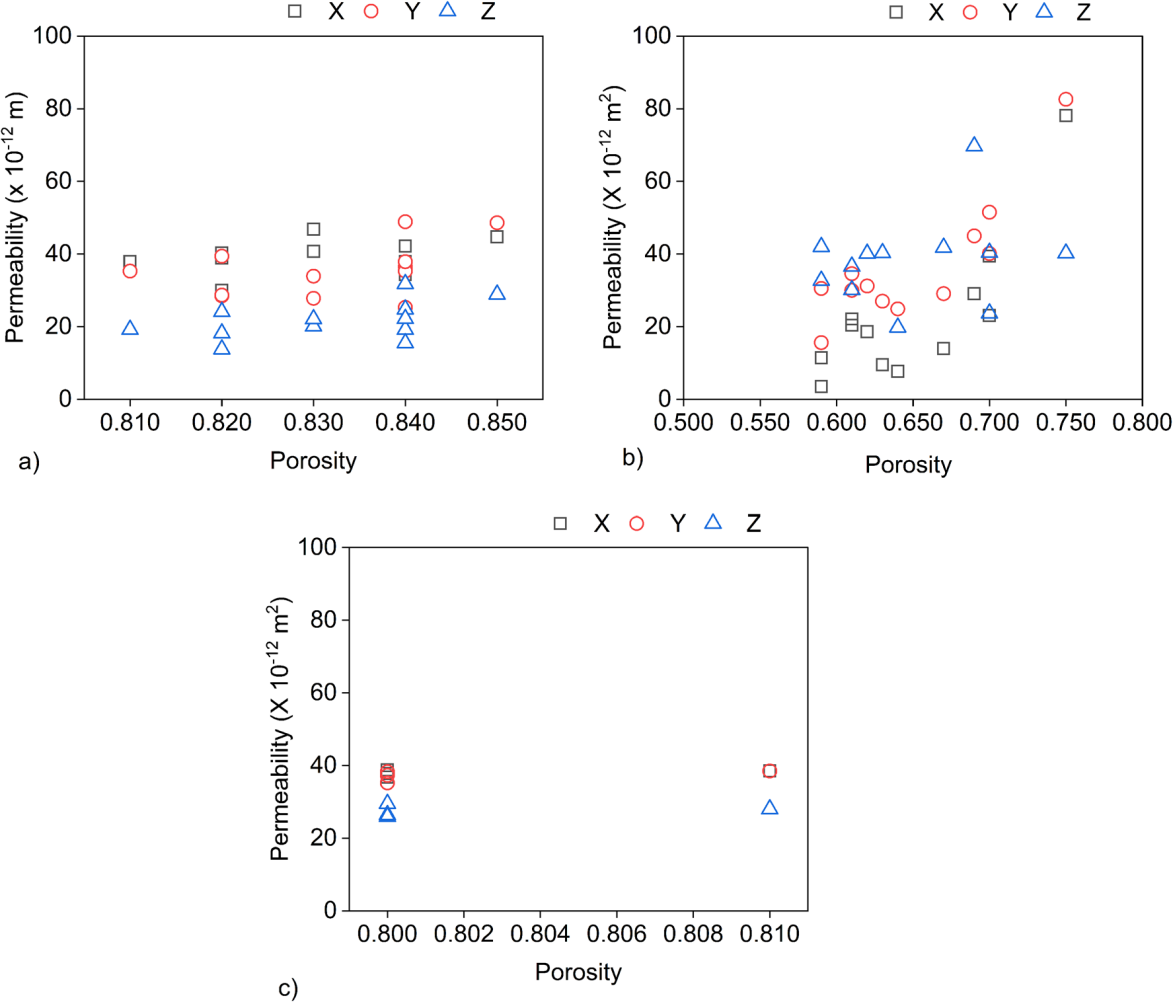
Computed in-plane (x and y) and through-plane (z) permeability versus porosity values across segmented sections of the simulated (**a**) Toray TGP-H-060, (**b**) Sigracet SGL 25 BA, and (**c**) AvCarb MGL 370 GDLs.MGL shows little variation since the porosity is almost homogeneous in all four sections. TGP-H and SGL 25-BA each have twelve sections.

Porosity calculations showed heterogeneity in TGP-H, even though minimal, across different GDL sections. However, it is worth noting that the variation seen was primarily observed across the sections in Fig. 2a categorised as 1 to 4 (That is TC1–TC4, TR1–TR4, and TL1–TL4). The observed heterogeneity in porosity within the studied GDL sections is consistent with previous findings in the literature^24^, where the GDL was also divided into sections before simulation. This could be attributed to many factors, such as fibre arrangements and the distribution of PTFE loading in the GDL. The minimum porosity value observed was 0.81 in TL3, and the maximum was 0.85, with an average porosity of 0.83 in this GDL. The porosity value of the woven cloth GDL was determined to be 0.82. A mean porosity of 0.80 was obtained in the MGL, with little to no variation across all four regions of the simulated GDL, as illustrated in Fig. 6.

On the other hand, it is important to emphasise the substantial heterogeneity in porosity values inside the SGL, as this greatly affected the calculated permeability results. For instance, along the channel, the region with the highest porosity of 0.75, SGLC1, exhibits in-plane x and y direction permeability of ${78.1 \times 10^{-12}}$ $\textrm{m}^2$ and ${82.6 \times 10^{-12}}$ $\textrm{m}^2$, respectively, whereas the through-plane permeability measures ${40.2 \times 10^{-12}}$ $\textrm{m}^2$. Section SGLC4, with a porosity of 0.69, displays a higher through-plane permeability when compared to SGLC1. Interestingly, this trend is reversed when examining their in-plane permeability. Following a visual examination of these layers, it was observed that the density of the GDL fibres decreased from Region 4 to Region 1 with a porosity value of SGLC1 identical to that of the SGL GDL before it was put into the cell. This indicates that apart from the porosity, the fibre density could also influence the permeability, and is in agreement with literature^62^. Nevertheless, as the fibre density is beyond our scope, we did not investigate further. Furthermore, it seems this increase in density is due to the presence of binders, which shows that the binder significantly influences the topology of the SGL microstructure and changes its morphology from that of traditional fibrous materials. Conclusively, the variation in porosity distribution among different regions significantly impacts their distinct permeability properties. However, despite the proposals made in previous research^24–26^, no distinct linear association was observed between porosity and permeability for the simulation scenarios in this investigation. In our findings, we propose that the correlation between porosity and permeability was influenced by the presence of binders in the SGL and TGP-H.

In summary, the calculated average permeability for the respective GDLs in the in-plane and through-plane directions and the mean porosity are given in Table 3. Note that the values in the table for TGP-H and SGL only include the channel area, while the mean value, including areas under the ribs, can be found in Fig. 5. Since the resistance to bulk movement of air through a GDL to the catalyst layer is characterised by its through-plane permeability^2^, an increase in this improves the performance of the GDL. At the same time, the in-plane permeability indicates the convective flow of the reactant gases through the GDL parallel to the flow channels of the bipolar plate. The through-plane permeability of a toray GDL has been noted to increase with increased porosity^6^. This explains why the through-plane permeability values in our GDLs are seen to be considerably larger than previous studies, with lower porosity than ours^17,62, 64^. On the other hand, in-plane permeability of particular GDL increases with thickness due to increased porosity, according to^2^. In contrast, our work did not observe this trend. The observed heterogeneity in porosity within the studied GDL sections is consistent with previous findings in the literature^24^.

**Table 3:** Computed upscaled absolute permeability and porosity of the respective GDLs studied and their respective thickness without rib compression.

GDL Studied	Permeability (10 ^−12^ m^2^)			Porosity	Thickness ($\upmu$m)
	x-Direction	y-Direction	z-Direction		
SGL 25BA	${42.4}$	${54.8}$	${43.5}$	0.71	168
TGP-H 060	${40.9}$	${43.7}$	${26.8}$	0.84	176
MGL 370	${38.0}$	${37.3}$	${27.7}$	0.80	307.5
W1S1011	${13.6}$	${11.6}$	${9.32}$	0.82	97.86

### Effects of compression on GDL’s pore size and permeability

Images of TGP-H and SGL were taken operando; hence, to examine the influence of rib compression on GDL performance, the regions beneath the ribs and channels of Toray TGP-H 060 (TGP-H) and Sigracet SGL 25BA (SGL) GDLs were separately analysed. This is necessary to investigate the influence of cell assembly on GDL permeability.

From earlier sections about the GDL simulation domain, it has been observed that the GDL thickness for TGP-H changes from 176 $\mu$m under the channel to 170.5 $\mu$m under the ribs and for SGL, this changes from 168 $\mu$m under the channel, to 135 $\mu$m under the ribs. These provide an estimated compression of 3.1 % and 19.6 % undergone by TGP-H and SGL, respectively. In the subsequent sections, we examine the impact of this compression on the distribution of pore sizes, permeability, and porosity.

#### Pore size distribution

**Figure 7:**
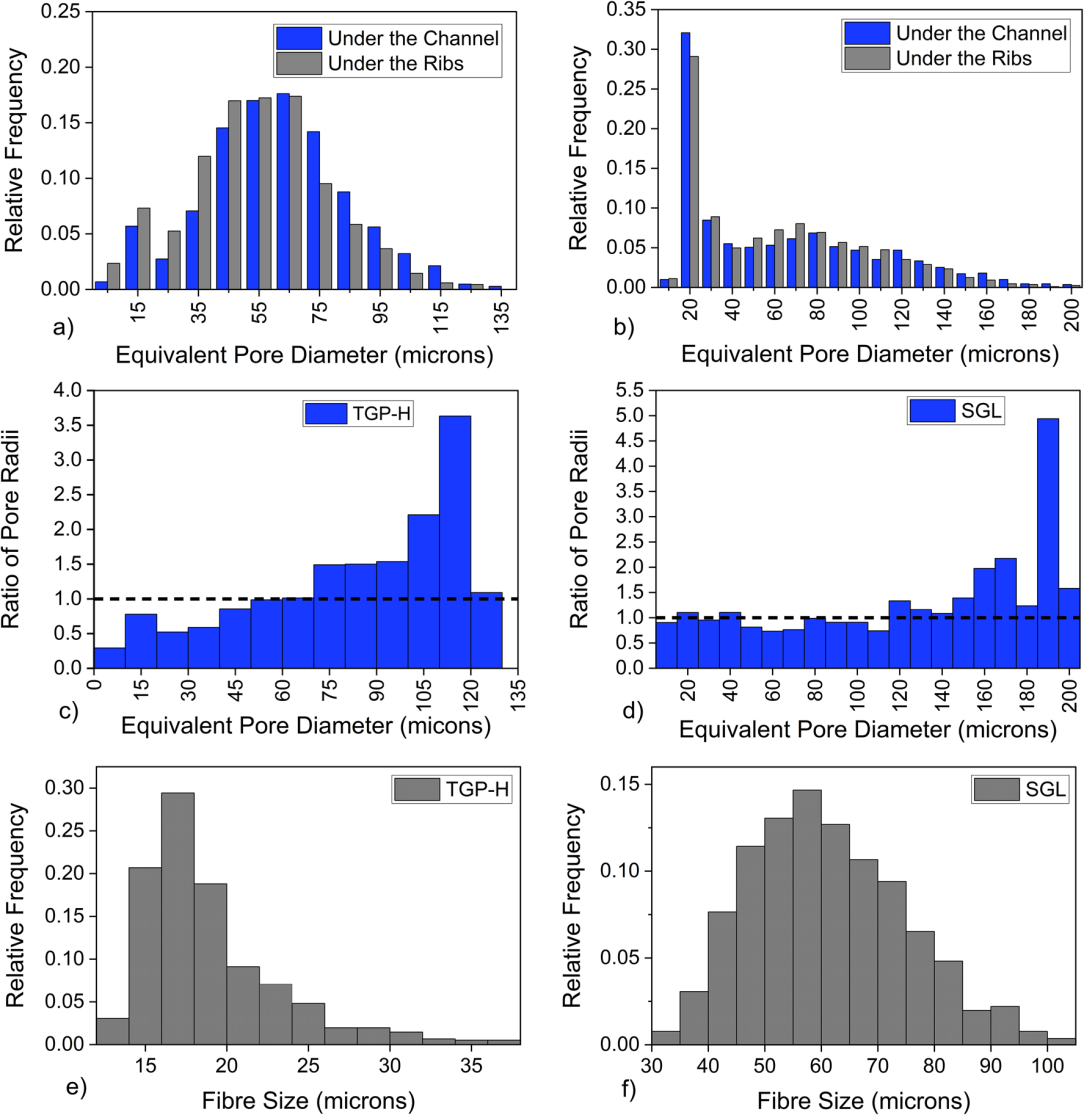
Pore diameter distribution of Toray TGP-H-060 (**a**) and Sigracet SGL 25BA (**b**), distribution of the ratio of the frequency of pore size for sections under the channel to the sections under the ribs for Toray TGP-H-060 (**c**) and Sigracet SGL 25BA (**d**) and fibre size distribution of Toray TGP-H-060 (**e**) and Sigracet SGL 25BA (**f**).

Figure 7a, b show the equivalent diameter ($D_{eq}$) of pores found in the areas beneath the ribs and the channel for TGP-H and SGL, respectively. As depicted, there are discernible disparities in the distribution of pore sizes. In TGP-H, a standard Gaussian distribution characterises the pore size distribution, showcasing an average pore diameter of 66 $\upmu$m. This distribution is similar to that found in previous studies^28,65^, where their average pore size was found to be 42 $\upmu$m and 53 $\upmu$m respectively. In SGL, the distribution is slightly different, with a greater concentration of pores that are predominantly smaller in size. Specifically, the most frequent pore size beneath the ribs and channels registers an $D_{eq}$ of approximately 20 $\upmu$m. The studied SGL has an average pore size of 63 $\upmu$m. The values of the average pore size in TGP-H and SGL align with literature where the pore size of GDL is recorded to be between 10 and 100 $\upmu$m^2,64^. Upon careful examination of the distribution of pore sizes, it is imperative to ascertain the occurrence and nature of any compression phenomena, thereby understanding how they appear and how widespread they are in the materials.

Figure 7c, d show the ratio of the frequency of pore size for sections under the channel to the sections under the ribs for TGP-H and SGL, respectively. The horizontal dashed line shows where the number of pores with a particular $D_{eq}$ is the same in both sections. Above the dashed line, show which pore class can be found more in the areas under the channel. Below the dashed line, in areas less than one, shows that the pores with such sizes are more frequent in the areas under the ribs. Despite the seemingly lesser effect of rib compression on TGP-H in terms of thickness when compared with SGL, Fig. 7c depicts significant compression from the ribs took place in the TGP-H GDL, leading to a four-fold increase in the number of smaller pores found under the ribs. In contrast, the number of larger pores here decreased substantially. Despite the observable compression in the SGL GDL, as seen from the change in thickness, the frequency of pores size in areas under the ribs and under the channel is almost identical (see Fig. 7d). Moreover, there is only less than a twofold increase in smaller pores in the areas under the ribs. This could be because the SGL pore distribution already showed a greater concentration of smaller pores than large pores. Furthermore, when the compression in SGL from Fig. 6b, d was analysed, it seemed evident that a number of the pores (including 20 $\upmu$m) could have been reduced or even eliminated (pore size 125 $\upmu$m and above) by compression. The presence of smaller pores under the ribs could mean that there is increase in gas transport pathways in those areas, as gas would preferentially go through smaller pores in a hydrophobic GDL.

The fibre size (diameter) distributions of TGP-H and SGL employed in our study are presented in Fig. 7e, f, respectively. The average fibre size of TGP-H is 18 $\upmu$m, whereas SGL has an average fibre size of 66 $\upmu$m. These GDLs are coated, hence the variation with those in literature with sizes 7 $\upmu$m^42,66^ and 9 $\upmu$m^42^ for Toray TGP-H-60 and SGL 25BA, respectively. Nevertheless, our findings align with the broader range of fibre diameters, which spans from 10 $\mu$m to 100 $\mu$m for both GDLs^1,2, 8^. Although the SGL has a bigger fibre size than the TGP-H, it underwent greater thickness reduction due to compression. This phenomenon may be attributed to their individual fibre densities. Although we did not specifically measure the densities of the GDL fibres, based on visual observation, TGP-H appeared to have a higher density than SGL. This is substantiated by other studies^67,68^. In was shown that the high resistance to deformation shown by Toray 60 was attributed to its relatively high-density carbon fibres compared to those of SGL-24BA^68^. Their study revealed a density value of 0.44 g $\textrm{cm}^{-3}$ for Toray, which had a thickness of 190 $\mu$m and a porosity of 0.78. The SGL-24-BA material was found to have a thickness of 256 $\mu$m and a porosity of 0.74. Its density was measured to be 0.28 g $\textrm{cm}^{-3}$^68^.

#### Impact of compression on porosity and permeability of GDLs

Regarding the impact of compression on porosity values, the observed values in TGP-H showed minimal deviation, with average porosity measuring 0.83 beneath the ribs and 0.84 beneath the channel. Conversely, the average porosity values in SGL were 0.62 under the ribs and 0.71 beneath the channel. Notably, the initial porosity of the SGL used in this simulation was 0.75 before assembly in the cell^46^. This indicates that the compression of GDL experienced by areas under the ribs is a secondary compression, following an initial compression from the gasket that also affects the GDL under the channel area. Furthermore, the region under the channel SGLC1 has the highest porosity with a value of 0.75. In contrast, SGLR2 and SGLL2 have the smallest observed porosity (0.59). This suggests lower density in this region 1 due to less binder presence or less fibre density, leading to pore disappearance as a result of rib compression.

The variations in porosity values emphasise the substantial influence of compression forces applied by the ribs on the structural soundness and operational characteristics of the GDL. Within the context of TGP-H, the minimal deviation indicates a more evenly distributed compression, maintaining the fuel cell’s porosity and potentially improving its performance. On the other hand, the increased variability in SGL suggests stronger compression effects, which can impact the movement of fluids and gases within the cell. Understanding these variations is crucial for optimising GDL design and ensuring consistent performance under operational conditions.

The average single-phase permeability calculated in TGP-H and SGL GDLs taken under operational conditions was higher in areas beneath the channel, regardless of the directional aspect considered. We have calculated an estimated percentage change in the average permeability observed in the regions under the ribs compared to the average permeability of the regions beneath the channel caused by compression from the ribs. See Table 4. The data indicate that the compression of the GDL by the ribs has a more pronounced effect on the through-plane permeability of the TGP-H GDL compared to the SGL GDL. This differential impact suggests that the regions under the ribs in the TGP-H GDL are more susceptible to the formation of water clusters due to rib compression, in contrast to the SGL GDL. Figure 5b (and in the SI Fig. S3b) underscores a substantial disparity in in-plane permeability between areas under the channel and those under the ribs. The disparity is particularly conspicuous in divided sections 1 (SGLR1, SGLL1, and SGLC1), where the in-plane permeability in the channel region (SGLC1) significantly surpasses that in the rib regions (SGLR1 and SGLL1). This observation aligns with findings in^68^ and could potentially be elucidated by factors such as solid volume fraction (SVF) or the density of the SGL, which also significantly affect porosity. For deeper insights into the relationship between SVF and permeability, references such as^34,68^ provide valuable context. Consequently, this suggests that in GDLs with lower fibre density or sparser SVF, rib compression disproportionately influences in-plane gas permeability compared to through-plane gas permeability. Moreover, the identical porosity values of the SGLL2 and SGLR2 sections which still yielded entirely different permeability values, especially in the in-plane x-direction of SGLL2 (see Fig. 5b, and Fig. S3b measuring $3.5 \times 10^{-12}$ $\textrm{m}^2$, suggest the presence of dead-end pores and show that apart from the porosity, pore size distribution and pore connectivity after compression plays a vital role in GDL permeability. Slight increases in porosity (0.63 and 0.64) in SGLL1 and SGLR1 also correspond to reduced permeability. These disparities suggest the presence of dead-end pores in the SGL GDL, impacting permeability despite similar porosity levels. This behaviour observed in the SGL GDL aligns with prior findings (e.g.,^69^) regarding anisotropic behaviour in the SGL GDL. Further investigation using advanced methods like microscopy is crucial for understanding these variations, which are essential for optimising the GDL’s performance in applications reliant on precise transport control.

**Table 4:** Estimated percentage changes in permeability in areas under the ribs and channels due to GDL compression by the ribs.

GDL type		TGP-H 060	SGL 25 BA
% Compression		3.1	19.6
Total change in permeability (%)	x	2.6–11	68 ± 0.2
	y	23–33	48–51
	z	23–34	17–19

The GDL thicknesses in the channel and rib areas were 176 $\upmu$m and 170.5 $\upmu$m for Toray TGP-H 060, and 168 $\upmu$m and 135 $\upmu$m for SGL 25 BA,respectively.

### End-point relative permeability values

Two-phase flow conditions in porous media can give rise to a preferential flow pathway. Additionally, studies have demonstrated that even in the absence of heterogeneity in the porous medium, the uniformity of fluid access to the pores is not consistent and is influenced by the saturation topology and pore-scale velocity field^70^. Two-phase flow in porous media at the Darcy scale is explained by relative permeability curves as a function of saturation. Relative permeability curves have two major parameters: the curvature of the curves and end-point saturations. They are very important parameters for setting up Darcy-scale models representative of a given porous medium.

This section reports the end-point relative permeabilities at different flow rate for air-water fluids in the GDLs. The simulated end points have been reported for both drainage and imbibition processes. Due to the hydrophobic nature of the GDLs under study, water injection represents the drainage process, while air injection stands for imbibition. A buffer zone was incorporated into the porous domain to facilitate uniform injection into the GDL. Although this method does not replicate in-situ operando conditions in fuel cells with MPL, it was employed to enable fluids to select their preferred pathways based on pore size and wettability.

**Figure 8:**
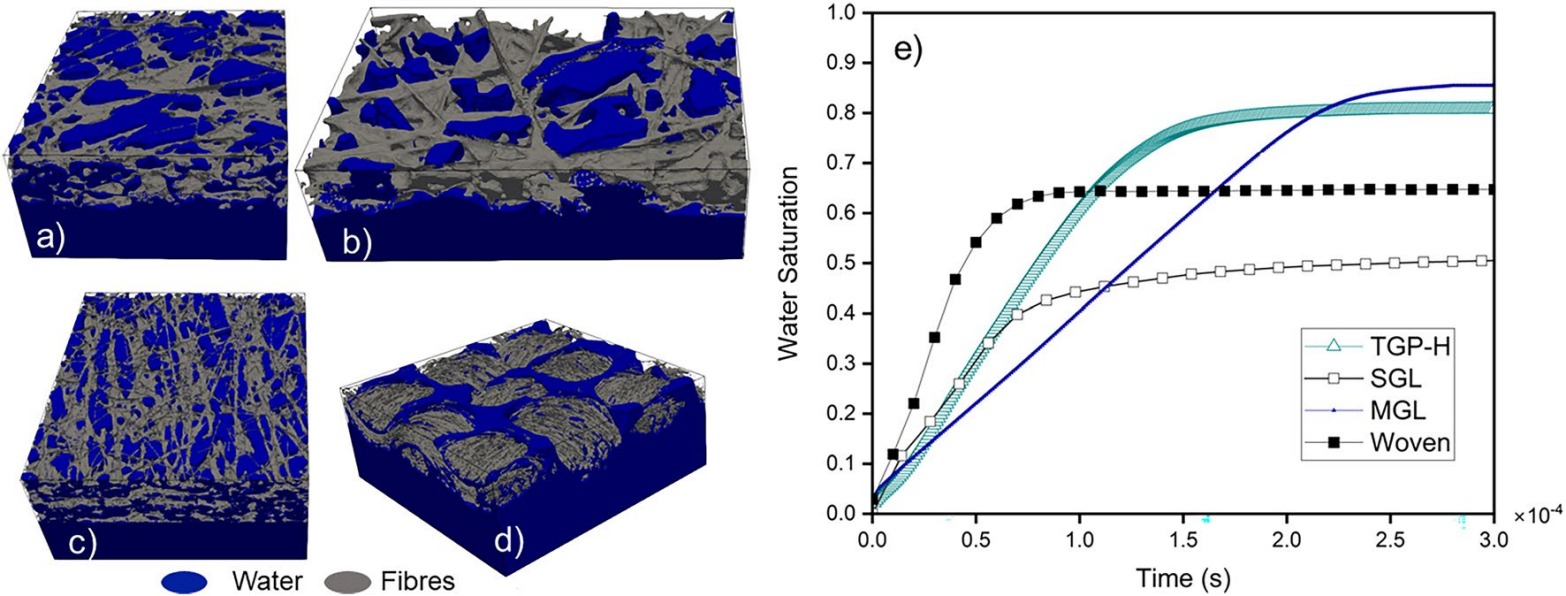
Steady-state saturated Toray TGP-H-060, Sigracet SGL 25 BA, AvCarb MGL 370, and the woven GDLs depicted as (**a–d**) respectively at water injection velocity of 1 $\mathrm{m \, s}^{-1}$. Plot (**e**) illustrates saturation versus time for all four GDLs at a velocity of $\mathrm{1 m\, s}^{-1}$.

In Fig. 8, the drainage process for the four studied GDLs (Toray TGP-H-060, Sigracet SGL 25 BA, AvCarb MGL 370, and the woven GDL) is depicted as (a–d), respectively at the water injection rate of $1 \text {m}^{-1}$. Each GDL was allowed to reach full saturation within $3 \times 10^{-4}$ s simulation time units, as demonstrated in Fig. 8e).

Interestingly, each GDL morphology contributed to the total saturation and endpoint relative permeability value. For instance, MGL has a total water saturation of 0.854 and an endpoint water relative permeability of 0.125, TGP-H has a total water saturation of 0.807 and an endpoint water relative permeability of 0.132, SGL has a total water saturation of 0.52 and an endpoint water relative permeability of 0.053, and woven GDL has a total water saturation of 0.659. Upon closer examination and comparative analysis of the fibre and pore size distributions in each carbon fibre GDL, distinct patterns emerge. As depicted in Figure S2 in the supplementary information, the SGL GDL exhibits considerably larger fibre dimensions in contrast to the TGP-H and MGL GDLs, primarily attributed to the influence of binders. Additionally, the observed scarcity of fibres suggests a lower fibre density relative to the other two GDL types. Consequently, the combination of larger fibres with reduced density contributes to the formation of prominent large pores, as illustrated in Fig. 7b and Fig. S2b, although present in smaller quantities relative to smaller pores. We propose that this phenomenon facilitates the preferential flow of water through the larger pores within the SGL GDL, resulting in accelerated achievement of steady-state saturation compared to the TGP-H and MGL GDLs. Nonetheless, the overall saturation level within the SGL GDL remains comparatively lower due to the predominance of smaller pores over larger and also dead-end pores (Fig. S3b the supplementary information). The woven GDL was exempted from the plots because of its material and morphology. However, Fig. S3c shows that water flows freely in the open areas between the tows rather than through the tows as expected. This free passage between the tows makes the GDL have a lower saturation and reach a breakthrough and steady-state saturation faster than the MGL or TGP-H.

During actual operations, it is desirable to have lower water saturation and a quicker water breakthrough saturation from the GDL to the flow channels. However, it is crucial to have interconnected pores in order to boost permeability and improve air convection and diffusion.In most cases, the microporous layer (MPL) is incorporated into the MEA configuration to create a gradient of porosity in the through-plane direction and improve permeability. Toray and MGL will undoubtedly gain advantages from utilising MPL to enhance the porosity gradient and augment the through-plane permeability. In addition, cracks in the MPL connected to these GDLs can act as conduits that create specific routes for water, limiting the spread of liquid water. This has the potential to decrease the duration required to achieve a breakthrough and significantly diminish the saturation of these GDLs at the endpoint. While the woven GDL and SGL take approximately the same amount of time to attain breakthrough saturation, the woven GDL has a higher saturation point because it has a greater density in the tows. Therefore, incorporating an MPL into this GDL will be advantageous. Based on our investigation, the SGL is the sole Gas Diffusion Layer that exhibits a through-plane permeability higher than its in-plane permeability. In conjunction with its large pores, this attribute expedited the flow to achieve breakthrough and steady-state saturation more rapidly than other GDLs. Moreover, its endpoint saturation is significantly lower compared to the other GDLs. This is because the water can pass through the wide pores and reach a point where it breaks through. However, we suggest incorporating MPL into this GDL if the primary objective is to minimise the impact of compression. Minimising compression can decrease the number of dead-end pores in the GDL, resulting in improved permeability.

Furthermore, it is observed during the drainage process that water preferentially passes through all channels at a high velocity of $\mathrm{1 m\, s}^{-1}$, filling the larger pore throats first, followed by the smaller ones due to capillary pressure and the hydrophobic nature of the porous GDL. At reduced velocities, water preferentially pushes through the larger pores and not the smaller pores heading towards the outlet. This phenomenon is the same in all GDLs and is depicted using the TGP-H GDL as seen in Figure S2a in the supplementary information, and leads to a lower water relative permeability and water saturation in each GDL at lower velocities.

**Figure 9:**
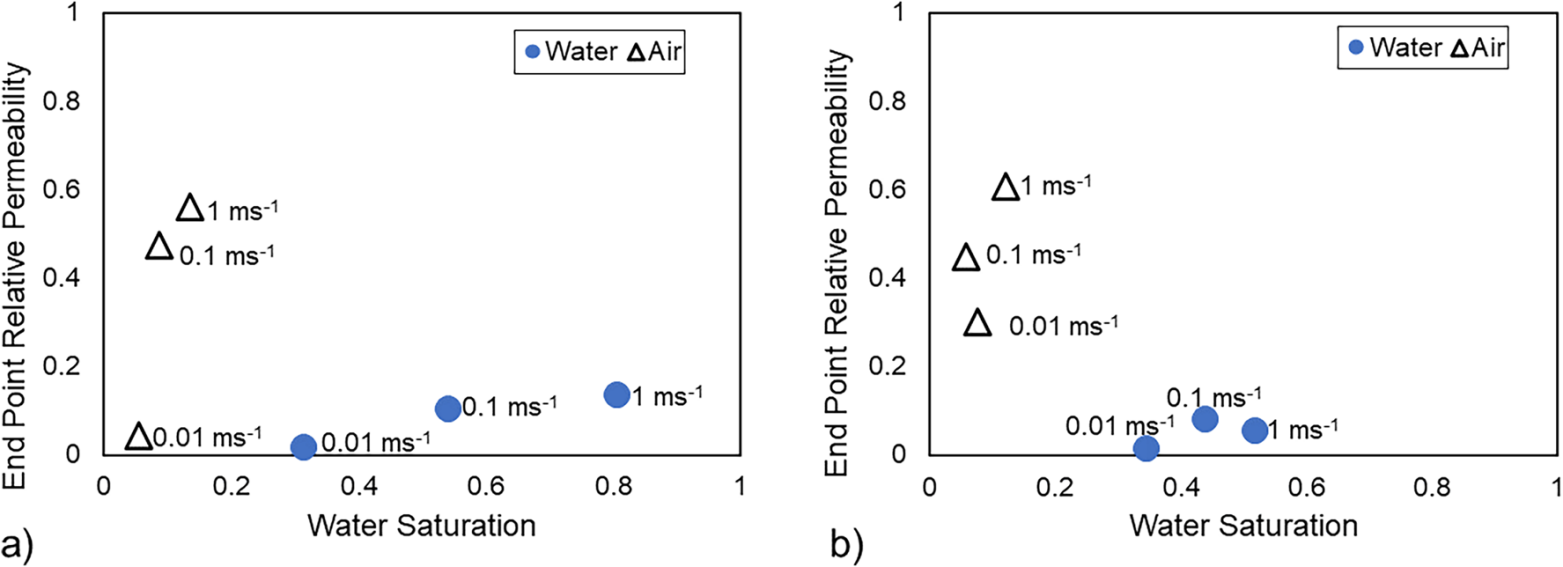
End-point relative permeability values of water and air for Toray TGP-H-060 (**a**) and Sigracet SGL 25 BA (**b**), respectively at three different injection rates. The impact of dynamic effects on the variability of the air end-point relative permeability is significant. Note water is the non-wetting phase in these simulations.

The influence of velocity on the end-point relative permeability and water saturation in GDLs was investigated. In Fig. 9, we observe the end-point relative permeability values of water and air for two GDLs: Toray TGP-H-060 (a) and Sigracet SGL 25 BA (b). It was observed that decreasing velocity led to reductions in both end-point relative permeability and water saturations of water and air. Notably, the impact of velocity changes was more pronounced in the air end-point relative permeability. Furthermore, despite a decrease in water saturation in the GDL at a velocity of 0.1 $\mathrm{m \, s}^{-1}$, an increase in the end-point relative permeability was observed. These observations underscore the influential role of porous media topology and wettability in shaping displacement patterns, transcending fluid properties and flow conditions.

Moreover, water clusters are observed in all GDLs during imbibition simulations with an air velocity of 1 $\mathrm{m \, s}^{-1}$. This indicates that despite effective water removal methods from the GDL, there are still phases of trapped water within it. This issue could potentially be addressed by drying techniques as demonstrated in prior research^71^ or by adjusting the air humidity to facilitate the removal of stagnated or trapped water through evaporation.

It is also observed during imbibition simulation that water wicks into the buffer zone from where the air is fed. This phenomenon was more pronounced as the velocity reduced and caused observed fluctuations in the remaining water saturation profile of the Toray TGP-H GDL. Furthermore, especially observable in the SGL GDL, the remaining water saturation increases slightly more than that of the previous higher velocity, as the amount of water that wicks into the buffer zone increased. We postulate that the low capillary number of air within the GDL, and the wettability of the buffer zone, could be the reason for this increase in water saturation and movement of water to the buffer zone respectively.

Under operating conditions, this buffer zone could be the flow channel. This would mean that depending on the wettability and type of the channel and GDL characteristics, liquid water could be well up in the channel and cause increased saturation in the GDL. It has been postulated that adding baffles to a flow channel in a system that uses a reduced porosity and permeability GDL could increase the water removal efficiency^72^. Hence, it could be beneficial to use baffled flow channels with a GDL like the woven GDL with lower permeability or the SGL with lower porosity values. Furthermore^2^, recorded that GDLs with high in-plane permeability will be optimised when used with an interdigitated flow field as a result of gas convection improvement but will not do well with serpentine or parallel flow field designs. Our results show that MGL and TGP-H fit into this description.

## Conclusions

The main premise of this paper was to comparatively analyse the permeability in various commercial GDLs - Toray TGP-H 060 (TGP-H), sigracet SGL 25 BA (SGL), AvCarb 370 MGL (MGL), and CeTech W1S1011, a woven GDL. Moreover, we delineated the impact of GDL compression on permeability and flow dynamics in the distinct zones beneath the ribs and the channel for both SGL and TGP-H. Furthermore, we aimed to investigate the end-point relative permeability data of the analysed GDLs.

We have shown that in all GDL studied, the through-plane permeability is lower than in-plane, except in the SGL where the opposite is true. This suggests that the fibres in the SGL are aligned differently from those of the TGP-H and MGL GDL. In addition, it reinforces the notion that fibre alignment plays a crucial role in determining permeability. Furthermore, this study demonstrated that the in-plane permeability in TGP-H and SGL GDL, decreased with decreasing porosity due to rib compression. However, no direct correlation between porosity and permeability was found in the through-plane permeability.

Specifically, it has been shown that the permeability of the GDL is influenced by various factors such as porosity, rib compression, fibre size, and pore size distribution. These observations are in line with prior studies^2,31^. Additionally, the permeability at a given moment in the GDL is determined by combining all these properties, including fibre density and alignment. For instance, despite the SGL having a lower porosity than TGP-H and being more susceptible to rib compression, its permeability values are higher than TGP-H. This can be ascribed to additional characteristics such as its greater pore size, larger fibre size, and lower fibre density, hence affirming the multifaceted interplay of these parameters in determining the overall transport characteristics within the GDL.

Two-phase flow studies reveal that the GDL porosity, morphology, fibre and pore size distributions, and PTFE loading distribution can affect water distribution and hence the saturation of water in the GDL. Despite having just 5% PTFE loading in the SGL, its sparse fibre density compared to TGP-H, leads to deeper penetration of the loading within the fibres influencing the calculated fibre and pore size distributions. The outcome of this meant that SGL had a steep disparity in the number ratio of small pores to large pores compared to other GDLs and water preferentially passed through the larger pores making it attain steady state saturation faster. Pore size distribution in TGP-H and MGL GDL show similar Gaussian distribution pattern, making the water have an almost uniform pore penetration.

The findings of this study enhance our understanding of flow dynamics in different GDLs. This will help users make informed decisions when choosing a specific GDL based on their operational goals or the other components within the electrochemical device, such as the flow channel type or the option of having an MPL.

Furthermore, the knowledge of end-point relative permeability data is crucial in improving volume-average models, such as Darcy-based flow simulators, specifically tailored for simulating PEM Fuel Cell or PEM electrolyzer systems.

One potential limitation of this study is the uniform application of wettability throughout all GDLs, regardless of their varying PTFE concentrations. Nevertheless, the selected value is within the range of the real values documented in the literature on GDL wettability. Subsequent investigations can utilise the precise wettability measurements of individual GDLs. Another limitation is the substantial computational expense associated with employing this approach, leading to the use of a small-scale domain, and uniform velocity values. However, our findings still yielded valuable outcomes that are comparable to existing research.

### Supplementary Information

Supplementary Information.

## Data Availability

The dataset for the GDLs used can be found on the respective websites https://doi.psi.ch/detail/10.16907%2Fe40c68d6-b8ba-4e3d-85a4-17347c41f350 and https://doi.psi.ch/detail/10.16907%2Fa27fda1c-e6bb-47f0-ba14-31b407179756 for Toray TGP-H-060 and SGL 24 BA respectively. While those of MGL and the woven GDL can be found in https://www.digitalrocksportal.org/projects/462/origin_data/2538/ and https://urldefense.com/v3/, https:/doi.org/10.5281/zenodo.7470938__;!!PDiH4ENfjr2_Jw!F9lGmCB5_ku8PWUlycKwTlfngQe0Qx3Y5XhmIX4t7z8JX8U3DeeMn0FZs-kRvvdatxLj7j6NaslnB_cP_nLH-aYXVKwzWlyfRw$ respectively.
