# Supplementary material for: Characterisation of hydraulic properties of commercial gas diffusion layers: Toray, SGL, MGL, woven carbon cloth
Source: Sci Rep. 2024 Aug 13;14:18812. doi: 10.1038/s41598-024-68681-4 (PMC11322176; doi:10.1038/s41598-024-68681-4)
Supplement: Supplementary file 1 — Supplementary Information. [file 41598_2024_68681_MOESM1_ESM.pdf]

Supporting Information for "Characterisation of the hydraulic properties of commercial gas diffusion layers: Toray, SGL, MGL, woven carbon cloth"

Grace Esu-Ejemot Aquah[1], Daniel Niblet[2], Javad Shokri[1], and Vahid Niasar[1,*]

[1]Department of Chemical Engineering, University of Manchester, Manchester, M13 9PL, United Kingdom
[2]School of Engineering, Newcastle University, NE1 7RU, United Kingdom
[*]vahid.niasar@manchester.ac.uk

ABSTRACT

This study utilises computational fluid dynamics simulations with the *OpenFOAM* computational framework to investigate and compare the in-plane and through-plane permeability properties of four different gas diffusion layers (GDLs). Also the through-plane water and air relative permeability values and water saturations at different rates were simulated. Permeability analysis enhances our understanding of fluid flow, ways to decrease pressure loss in the GDL, and methods to enhance oxygen concentration at the catalyst layer interface through convection. The analysis reveals that the investigated GDL materials have spatial heterogeneity of porosity and permeability, especially in the Sigracet SGL 25 BA GDL. However, the porosity and permeability of the Toray TGP-H 060 and AvCarb 370 MGL GDLs exhibit less variations. The two-phase flow studies on GDL saturation show that at the same water injection flowrate, the AvCarb 370 MGL GDL has the largest remaining water saturation, with Sigracet SGL 25 BA GDL being the less saturated GDL among the four investigated GDLs. The compression from the ribs significantly affected the in-plane permeabilities of both Toray TGP-H 060 and especially impacted Sigracet SGL 25 BA GDL. This impact was expected as the pore size distribution varied significantly in the areas under the ribs versus the channel.

Table S1. A Table Showing the Dimension Size of GDLs Used for Single-Phase Simulation.

Materials	ID	Size of simulation domain (μm)
MGL	MGLL1	1333.5×1229×307.5
	MGLL2	
	MGLR1	
	MGLR2	
TGH	TR1	134.8×687.5×170.5
	TR2	
	TR3	
	TR4	
	TL1	165×687.5×170.5
	TL2	
	TL3	
	TL4	
	TC1	770×687.5×176
	TC2	
	TC3	
	TC4	
SGL	SGLR1	747×4497×135
	SGLR2	
	SGLR3	
	SGLR4	
	SGLL1	
	SGLL2	
	SGLL3	
	SGLL4	
	SGLC1	735×4497×168
	SGLC2	
	SGLC3	
	SGLC4	
Woven	W11	492×492×97.8

1 Mesh sensitivity analysis

In order to make sure that our results were independent of the mesh size, we conducted a mesh sensitivity analysis. Though this was carried out for every single layer simulated, we show the variation of permeability with cubic root of mesh size in Figure S1 (a), and slice of the resulting final mesh chosen for simulation to emphasise its quality after the use of snappyHexMesh (Figure S1 (b)).

Table S2. Sensitivity analysis of the mesh size to the results of the permeability calculations

Mesh Size	Cubic root	Permeability ($\times 10^{-12}$ m^2)	Percentage change
593,883	84.0556604	50	0.779
1,201,417	106.307668	34.3	0.217
2,986,293	144.004967	31.5	0.120
4,846,264	169.226753	28.4	0.010
5,092,439	172.044956	28.1	0

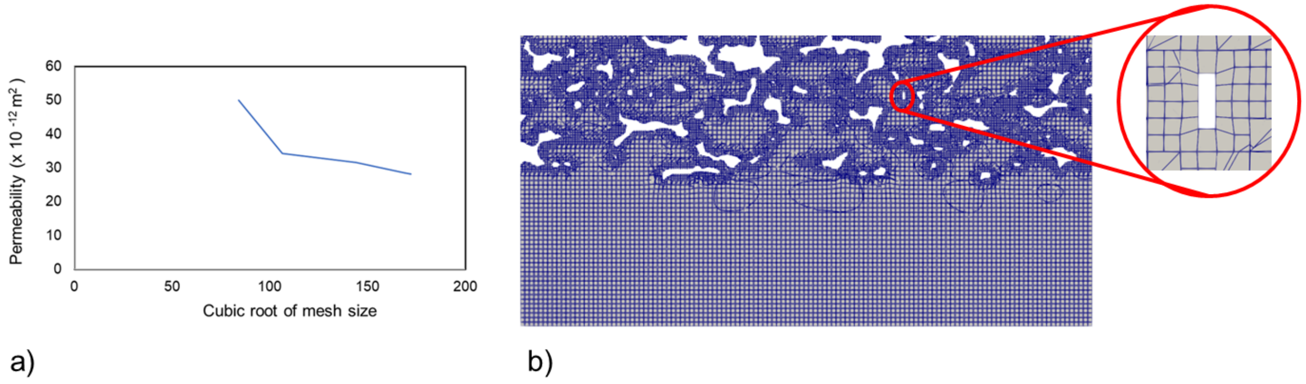

Figure S1. The mesh sensitivity analysis done on a single segment for Toray TGH-060 GDL (a) the variation of permeability with cubic root of mesh size, (b) the mesh quality of the final mesh

2 Method Validation

Using the Poiseuille equation, we calculated the velocity field of a 2D pipe both analytically and using OpenFoam SimpleFoam. The result of our analysis is given in the Figure S2 showing great match.

3 Comparison of fibre diameter and pores size distributions

The fibre diameter and equivalent pore diameter distributions of Toray TGP-H-060 (TGP-H), Sigracet SGL 25 BA (SGL), and AvCarb MGL 370 (MGL) carbon fibre GDLs were measured using a source code developed by[1,2] in MATLAB, and Avizo 2019 .1 respectively. This is depicted in Figure S3. The woven GDL was exempted because of its material and morphology.

4 Effect of compression on thickness, porosity and permeability

Table S4. The effect of ribs compression on the thickness of Toray TGP-H 060 and Sigracet SGL 25 BA

	TGP-H 060	SGL 25 BA
Uncompressed area (under the channel)	176 µm	168 µm
Compressed area (under the ribs)	170.5 µm	135 µm

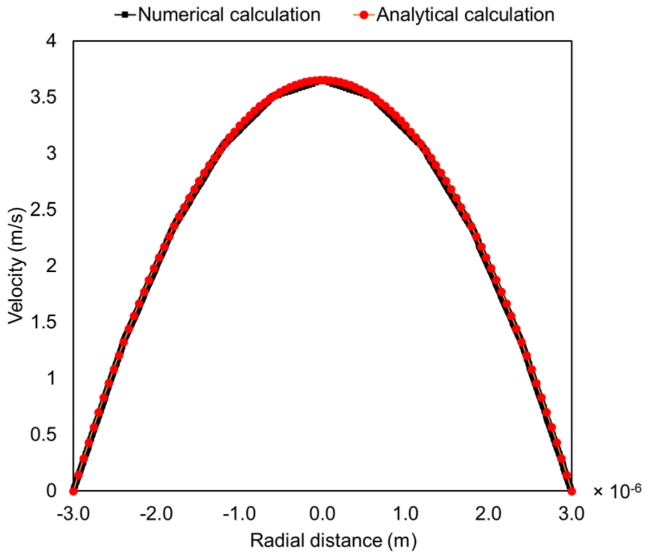

Figure S2. Velocity profile in a two-dimensional pipe

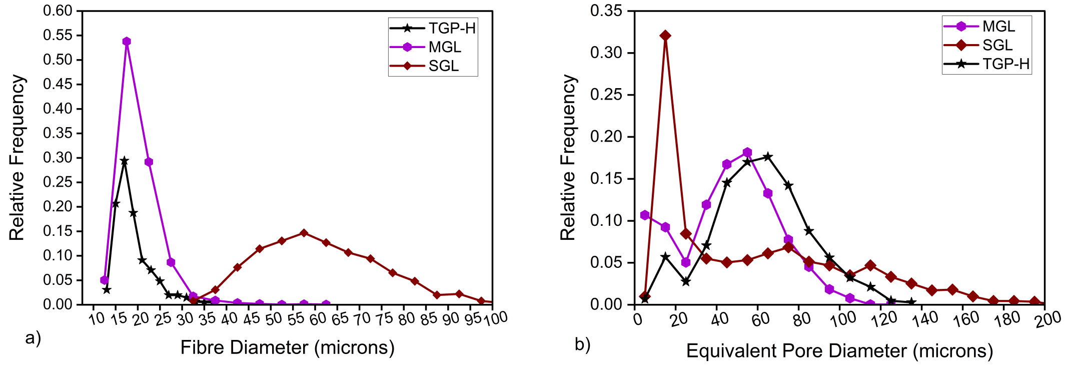

Figure S3. Comparative plots of the fibre diameter distributions (a) and pore size distributions (b) of Toray TGP-H-060 (TGP-H), Sigracet SGL 25 BA (SGL), and AvCarb MGL 370 (MGL).

5 Two-phase water flow in the gas diffusion layers

In addition to the influence of fibre and pore size distributions within the GDL on water imbibition and ensuing saturation levels, the velocity of the injected fluid and the GDLs' morphology also proved significant. This is evident from Figures S5 a) - c). While Figure S5 (a) shows the change in GDL steady-state saturation with decreasing velocity, in Figure S5 (b), the flow through a slice of the SGL GDL taken at two different locations can be seen. While the first figure shows free flow through the porous domain, the second figure highlights the presence of dead-end pores in the GDL. These dead-end pores however, do not seem to affect the overall flow within the GDL owing to the large pores in the GDL which allows the preferential passage of water out of the GDL, leading to reduced water saturation compared to other GDLs. Figure S5 (c) shows that water easily flows in the open areas between the tows that through the tows as is expected. This makes the woven GDL get to steady state saturation faster than the MGL or TGP-H.

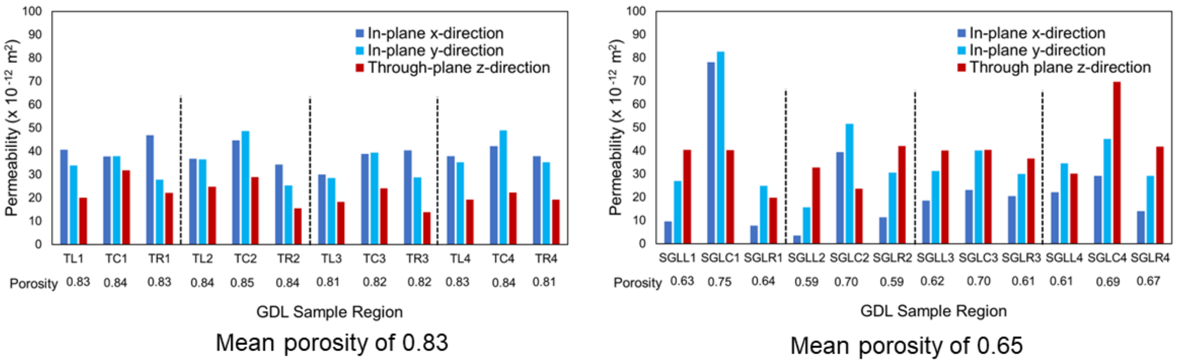

Figure S4. Effects of ribs compression on the permeability and porosity of the twelve sections of (a) Toray TGP-H-060 (b) Sigracet SGL 25BA. Four sections each of the left rib (TL and SGLL), channel area (TC and SGLC), and right rib (TR and SGLR). The dashed lines demarcate the sections (1-4)

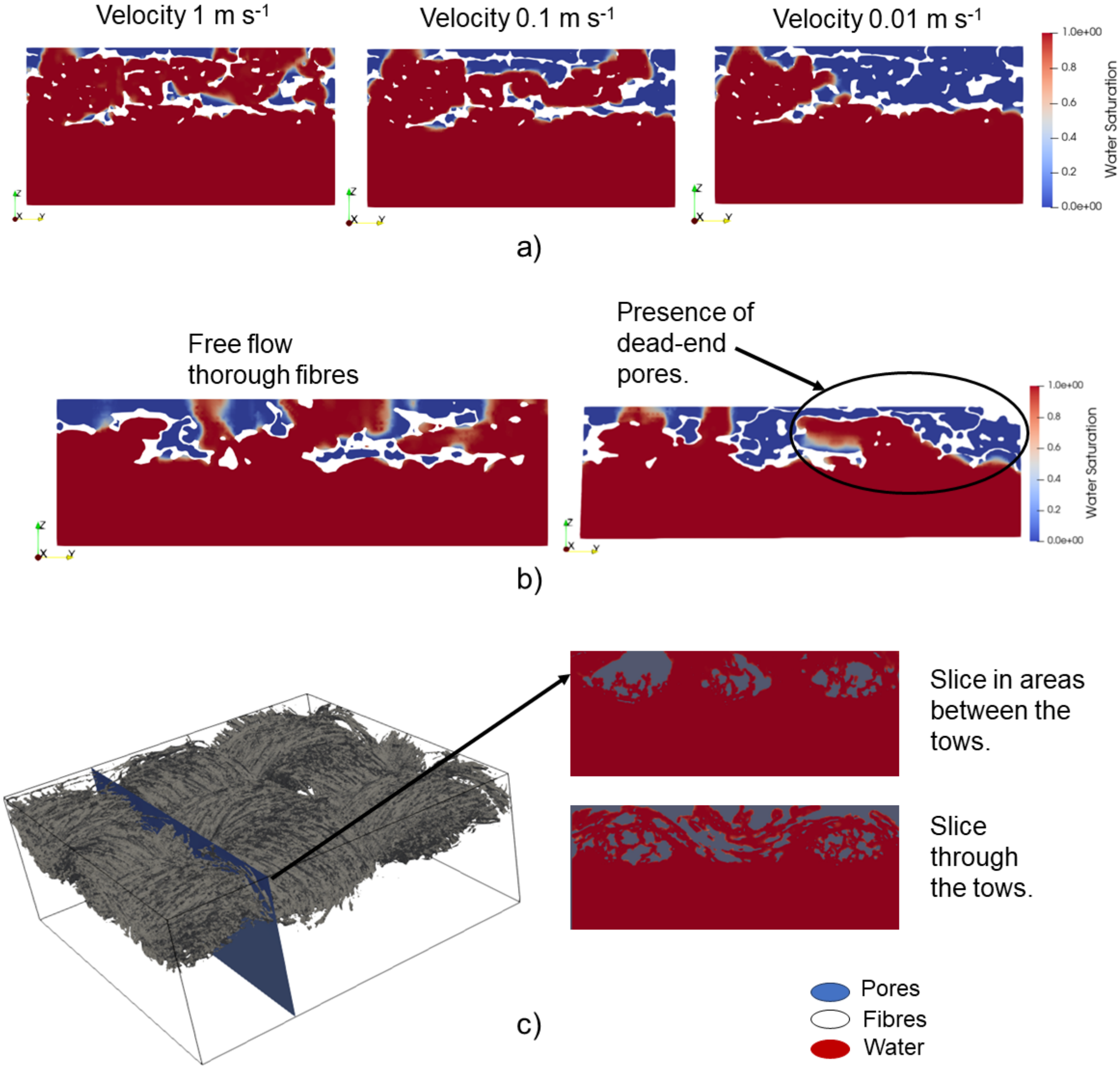

Figure S5. (a)The change in total saturation of the TGP-H GDL with changing flowrate (b) The SGL GDL showing areas of preferential free flow, and areas with high concentration of dead-end pores (c) The woven GDL showing variation in flow pattern in areas between the tows and through the tows.

References

1. Rabbani, A. & Salehi, S. Dynamic modeling of the formation damage and mud cake deposition using filtration theories coupled with SEM image processing. *J. Nat. Gas Sci. Eng.* **42**, DOI: 10.1016/j.jngse.2017.02.047 (2017).

2. Ezeakacha, C. P., Rabbani, A., Salehi, S. & Ghalambor, A. Integrated image processing and computational techniques to characterize formation damage. In *Proceedings - SPE International Symposium on Formation Damage Control*, vol. 2018-February, DOI: 10.2118/189509-ms (2018).
